# Genome Biology of *Actinobacillus pleuropneumoniae* JL03, an Isolate of Serotype 3 Prevalent in China

**DOI:** 10.1371/journal.pone.0001450

**Published:** 2008-01-16

**Authors:** Zhuofei Xu, Yan Zhou, Liangjun Li, Rui Zhou, Shaobo Xiao, Yun Wan, Sihua Zhang, Kai Wang, Wei Li, Lu Li, Hui Jin, Mingsong Kang, Baolige Dalai, Tingting Li, Lei Liu, Yangyi Cheng, Lei Zhang, Tao Xu, Huajun Zheng, Shiying Pu, Bofei Wang, Wenyi Gu, Xiang-Lin Zhang, Geng-Feng Zhu, Shengyue Wang, Guo-Ping Zhao, Huanchun Chen

**Affiliations:** 1 Division of Animal Infectious Disease, State Key Laboratory of Agricultural Microbiology, College of Veterinary Medicine, Huazhong Agricultural University, Wuhan, China; 2 Shanghai - MOST Key Laboratory of Health and Disease Genomics, Chinese National Human Genome Center at Shanghai, Shanghai, China; 3 State Key Laboratory of Genetic Engineering, Department of Microbiology, School of Life Science, Fudan University, Shanghai, China; 4 National Engineering Center for Biochip Research at Shanghai, Shanghai, China; 5 Laboratory of Molecular Microbiology, Institute of Plant Physiology and Ecology, Shanghai Institutes for Biological Sciences, Chinese Academy of Sciences, Shanghai, China; Centre for DNA Fingerprinting and Diagnostics, India

## Abstract

*Actinobacillus pleuropneumoniae* is the etiologic agent of porcine contagious pleuropneumonia, a cause of considerable world wide economic losses in the swine industry. We sequenced the complete genome of *A. pleuropneumoniae*, JL03, an isolate of serotype 3 prevalent in China. Its genome is a single chromosome of 2,242,062 base pairs containing 2,097 predicted protein-coding sequences, six ribosomal rRNA operons, and 63 tRNA genes. Preliminary analysis of the genomic sequence and the functions of the encoded proteins not only confirmed the present physiological and pathological knowledge but also offered new insights into the metabolic and virulence characteristics of this important pathogen. We identified a full spectrum of genes related to its characteristic chemoheterotrophic catabolism of fermentation and respiration with an incomplete TCA system for anabolism. In addition to confirming the lack of ApxI toxin, identification of a nonsense mutation in *apxIVA* and a 5′-proximal truncation of the *flp* operon deleting both its promoter and the *flp1flp2tadV* genes have provided convincing scenarios for the low virulence property of JL03. Comparative genomic analysis using the available sequences of other serotypes, probable strain (serotype)-specific genomic islands related to capsular polysaccharides and lipopolysaccharide O-antigen biosyntheses were identified in JL03, which provides a foundation for future research into the mechanisms of serotypic diversity of *A. pleuropneumoniae*.

## Introduction

*Actinobacillus pleuropneumoniae* is a Gram-negative, facultatively anaerobic, non-motile, rod-shaped bacillus in the family of *Pasteurellaceae*, which is chemoheterotrophic possessing both metabolic patterns of fermentation and respiration. Members of the *Pasteurellaceae* are obligate parasites, primarily of mammals and birds, while *A. pleuropneumoniae* is the etiologic agent of porcine contagious pleuropneumonia, an infectious respiratory disease of swine, which causes important world wide economic losses in the pig industry. The pathogen invades the porcine tonsil and upper respiratory tract, and can be isolated from nasal cavities, tonsils, the middle ear cavity and the lungs of infected animals [1], [2]. Depending on the number of bacteria reaching the lung, the particular serotype of the infection and the immunological status of the host, the course of the disease can be divided into peracute, acute and chronic forms [1]. Peracute and acute cases usually show high mortality with pulmonary lesions characterized by severe oedema, inflammation, haemorrhage and necrosis, whereas the chronic form of disease is characterized by haemorrhagic, fibrinous and necrotic pleuritis, pericarditis and pneumonia [1].

The virulence of *A. pleuropneumoniae* is known to be associated with several factors, such as exotoxins, capsular polysaccharide (CPS), lipopolysaccharide (LPS), outer membrane proteins (OMPs), and iron uptake proteins [3]. In addition, some enzymes involved in anaerobic respiration also appear to play an important role in the virulence of *A. pleuropneumoniae* [4].

*A. pleuropneumoniae* has been classified into two nutritional biotypes: the biovar 1 is β-NAD-dependent while the less common biovar 2 is β-NAD-independent [3]. On the basis of their capsular and lipopolysaccharide antigens, 15 serotypes of *A. pleuropneumoniae* have been recognized, with variations in their virulence and regional distributions [5]. Serotypes 1, 5, and 7 are most commonly found in North America, whereas serotype 2 predominates in many European countries [3]. In China, the prevalent serotypes are 1, 3, 4, 5 and 7 [6].

To date, 8 complete genomic sequences are available within the family of *Pasteurellaceae.* Seven of them, *i.e.*, *A. pleuropneumoniae* L20 (accession no. CP000569), *Pasteurella multocida* Pm70 (AE004439) [7], *Haemophilus influenzae* Rd KW20 (L42023) [8] and 86-028NP (CP000057) [9], *H. ducreyi* 35000HP (AE017143), *Mannheimia succiniciproducens* MBEL55E (AE016827) [10] and *H. somnus* 129PT (CP000436) [11], are in the GenBank. While the complete sequence of *A. actinomycetemcomitans* is available from the web site of University of Oklahoma's Advanced Center for Genome Technology (http://www.genome.ou.edu). Among them, *A. pleuropneumoniae* L20 (serotype 5b) genomic sequence is the only one available in the *Actinobacillus* genus. In this study, we sequenced and analyzed the genome of *A. pleuropneumoniae* strain JL03, a Chinese field isolate of serotype 3. Together with the genomic sequence of L20, this information provides a firm foundation for future research into the genetic basis of metabolism, pathogenesis, virulence and serotype/biotype determination in *A. pleuropneumoniae*.

## Results and Discussion

### General features of the genome

**Figure 1:**
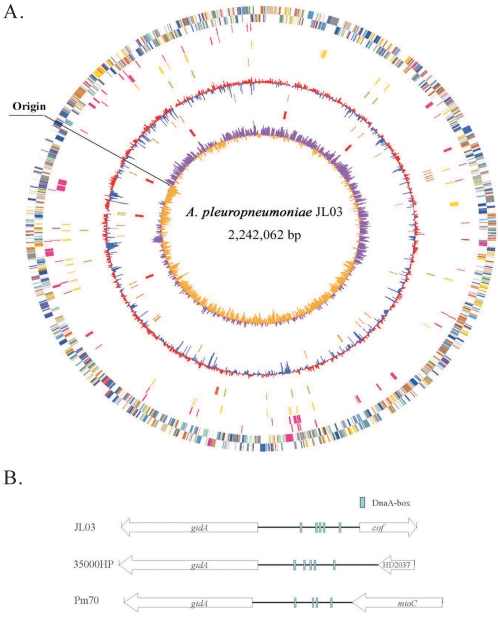
The characterizations of *A. pleuropneumoniae* JL03's genome and the *oriC* region. (A) Circular genome representation of JL03. Circles are numbered from 1 (outer circle) to 10 (inner circle). The circles 1/2 shows predicted CDSs on the plus and minus strand in JL03 color-coded by COG categories. All genes are colored according to biological functions: gold for translation, ribosomal structure and biogenesis; orange for RNA processing and modification; light orange for transcription; dark orange for DNA replication, recombination and repair; antique white for cell division and chromosome partitioning; pink for defense mechanisms; tomato for signal transduction mechanisms; peach for cell envelope biogenesis and outer membrane; deep pink for intracellular trafficking, secretion and vesicular transport; pale green for posttranslational modification, protein turnover and chaperones; royal blue energy production and conversion; blue for carbohydrate transport and metabolism; dodger blue for amino acid transport and metabolism; sky blue for nucleotide transport and metabolism; light blue for coenzyme metabolism; cyan for lipid metabolism; medium purple for inorganic ion transport and metabolism; aquamarine for secondary metabolites biosynthesis, transport and catabolism; gray for function unknown. Circle 3/4, the putative horizontal transferred genes in deep pink identified by SIGI-HMM on the forward and reverse strand. Circle 5, repetitive elements in yellow, above 200nt and cutoff value 1e-10. Circle 6, transposases in green and potential prophage genes in dark orange. Circle 7, mean centered GC content of JL03 genes (red: above mean, blue-below mean). Circle 8, tRNA genes in orange. Circle 9, rRNA genes in red. Circle 10, GC Skew plot (windowsize: 1000, windowoverlap: 500). (B) Genetic organization of the *oriC* regions in three representative organisms within the family of *Pasteurellaceae*: JL03, *A. pleuropneumoniae*; 35000HP, *H. ducreyi*; and Pm70, *P. multocida*.

The genome of *A. pleuropneumoniae* strain JL03 is composed of 2,242,062 base pairs (bps) with a single circular chromosome (Figure 1A). Referring to genomic coordinates of strain L20, the *dnaA* gene, designated APJL0001, was selected as the first gene of the JL03 genome. The putative replication origin (*oriC*) of JL03 chromosome was identified between two genes, *gidA* (APJL1688) and *cof* (APJL1689), based on GC skew and the presence of DnaA protein recognition sequences (DnaA-boxes) [12], [13] with typical gamma proteobacterium *oriC* features as what found in other genera of the *Pasteurellaceae* family (Figure 1B and Table 1).

**Table 1:** Comparison of the nucleotides conservation in DnaA-boxes of *oriC* among closely related organisms within the family *Pasteurellaceae*

Consensus sequence of DnaA-box	Length of *oriC* (bp)	A+T%	1	2	3	4	5	6	7	8	9	Number of DnaA-boxes
			T*	T	A	T	C	C	A	C	A	
*A. pleuropneumoniae* JL03	472	73.50%	100	100	80	100	100	80	80	100	100	5
*H. ducreyi* 35000HP	545	72.10%	100	100	100	100	100	100	60	80	100	5
*P. multocida* Pm70	489	72.10%	100	100	100	75	50	100	100	100	100	4

* Conservation (%) of nucleotide in DnaA-box of each species' *oriC*.

**Figure 2:**
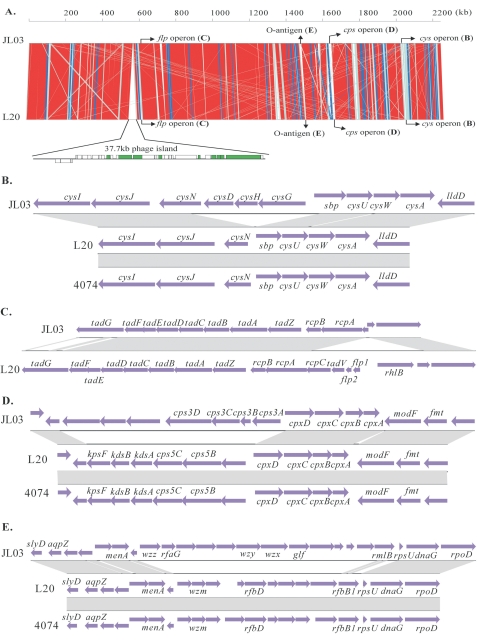
The schematic comparison of genetic organizations among three isolates of *A. pleuropneumoniae*. A co-linearity comparison diagram of the genomic organization at the nucleotide level between *A. pleuropneumoniae* strain JL03 and strain L20 (A). Color code stands for maximal length of those regions with highly homologous sequences between genomes: red, >10 kb; blue, 5–10 kb; cyan, 1–5 kb. The boxes in green represent phage-associated CDSs of L20. Besides the strain L20-specific prophage region illustrated below the linear genomic diagram as an enlarged drawing, four special genomic regions highlighted (B, C, D, E) were magnified in the corresponding panels. The genetic organizations of the *cys* operons (B), the CPS biosynthesis and export gene clusters (D), and the LPS O-antigen biosynthesis gene clusters (E) were compared among three isolates of *A. pleuropneumoniae*: JL03 (serotype 3), L20 (serotype 5b) and 4074 (serotype 1). Comparative genetic organization of the *flp* operons between JL03 and L20 is illustrated in panel C. Regions presented in gray represent highly homologous sequences. Blue arrows represent putative CDSs with either forward or reverse transcription directions.

The JL03 genome is approximately 1.4% smaller than that of strain L20 (2,274,482 bps). The genomic comparison of the linear organization at the nucleotide level between strains JL03 and L20 is presented in Figure 2A. Notably, strain L20 possesses a strain-specific genomic island of 37.7 kb encoding a number of phage-related proteins, which is absent in strain JL03.

There were eleven repetitive elements in the JL03 genome (designated JLRP1 to 11, hereafter) divided into several categories according to their coding sequences, *i.e.*, transposase, adhesin, elongation factor Tu and unknown proteins (Table 2). Among them, JLRP2, with its characteristic 25 bp inverted repeats in both ends, was presumed to be a novel insertion sequence element (IS) of the IS3 family. Submitted to the IS database (http://www-is.biotoul.fr), this sequence was designated ISAp12. In addition, a noncoding 2071 bp **c**lustered **r**egularly **i**nterspaced **s**hort **p**alindromic **r**epeats region (CRISPR) was identified in the vicinity of the *cas1* gene (APJL0215) that has been found adjacent to CRISPR loci in different bacteria [14]. This CRISPR is composed of an array of 28 bp direct repeats (DR) individually separated by 34 unique spacers of 32 bp or 33 bp. On the other hand, nine spacers in JL03's CRISPR all bear high sequence similarities with the corresponding sequences of plasmids from related bacteria (*A. actinomycetemcomitans*, *H. influenzae* and *H. ducreyi*). The inheritable feature of CRISPR spacers has been interpreted as evolutionary remnants derived from other extrachromosomal elements [15], and the CRISPR loci were successfully applied to studies in evolution, typing, and comparative genomics [16].

**Table 2:** List of repetitive elements in *A. pleuropneumoniae* JL03 genome

Repeat No.	Copies		Length (bp)	Identity (%)	Function for putative proteins encoded by genes within the repeats
	complete	partial			
JLRP1	2		2235	>99	serine-rich adhesin
JLRP2	7	4	1428	>97	transposase and inactivated derivatives
JLRP3	2		1252	100	elongation factor Tu
JLRP4	2		1201	100	hypothetical protein
JLRP5	7	2	1148	>99	hypothetical protein
JLRP6	3		1081	>98	hypothetical protein
JLRP7	2		758	>99	serine-rich adhesin
JLRP8	2		409	>86	outer membrane protein
JLRP9	2		404	>95	phosphoribosylglycinamide formyltransferase 2
JLRP10	2		388	>99	type I restriction enzyme EcoAI specificity protein
JLRP11	2		334	>82	hypothetical protein

Annotation of the JL03 genome is summarized in Table 3 and compared to those of strains L20 (*A. pleuropneumoniae*), Pm70 (*P. multocida*) and 35000HP (*H. ducreyi*). The entire JL03 genome has six ribosomal operons (16S-23S-5S rRNA) and an additional 5S rRNA. Sixty-three tRNA genes corresponding to the 20 common amino acids were identified in the JL03 genome. Four copies of tRNA-Ile and tRNA-Ala genes were located in the spacer regions between the 16S and 23S rRNA genes. A distinct selenocysteine tRNA gene containing the UCA anticodon was also identified. This tRNA gene is located adjacent to two genes (APJL1590, 1589) encoding L-seryl-tRNA selenium transferase (SelA) and selenocysteine-specific elongation factor (SelB), respectively. This kind of organization is the same as that found in *H. influenzae* strain 86-028NP [9].

**Table 3:** General features of the *A. pleuropneumoniae* JL03, L20, *P. multocida* Pm70 and *H. ducreyi* 35000HP genomes

GenBank accession No.	CP000687	CP000569	AE004439	AE017143
Strain	JL03	L20	Pm70	35000HP
Total length (bp)	2,242,062	2,274,482	2,257,487	1,698,955
Number of CDSs	2,097	2,012	2,014	1,717
Average length of CDS (bp)	941	976	997	842
CDS genome coverage	88.10%	86.37%	89.04%	85.12%
G+C%				
Total length	41.23%	41.30%	40.40%	38.22%
Protein gene	42.26%	42.33%	41.04%	38.74%
Intergenic region	33.63%	34.77%	35.26%	35.26%
Ribosome RNA				
16S rRNA	6	6	6	6
23S rRNA	6	6	6	6
5S rRNA	7	6	6	6
Number of tRNA	63	62	57	46
Number of tmRNA	1	1	1	1

The JL03 genome contained 2,097 potential CDSs with an average size of 941 bps, which in sum account for 88.1% of the whole chromosome. A graphical representation of CDSs by category and genetic characteristics of the JL03 genome are shown in Figure 1A. As shown in other completed microbial genomes, 18.3% of the CDSs were found to be similar to hypothetical proteins of unknown functions. The ortholog relationship between *A. pleuropneumoniae* and other species within the family *Pasteurellaceae* was consistent with their phylogenetic relationship based on the sequence analyses of 16S rRNA [17] and 50 highly conserved housekeeping genes [18]. Furthermore, protein homology comparisons demonstrated that *A. pleuropneumoniae* was closely related to *H. ducreyi* (1011 orthologous CDSs) but only distantly related to *H. somnus* (762 orthologous CDSs) (Table 4).

**Table 4:** Orthologs of predicted CDS of *A. pleuropneumoniae* JL03 compared with retrieved genomes

	numbers of CDS	Percentage
Homologues to *H. ducreyi* 35000HP	1011	48.2%
Homologues to *M. succiniciproducens* MBEL55E	960	45.8%
Homologues to *P. multocida* Pm70	900	42.9%
Homologues to *H. influenzae* Rd KW20	809	38.6%
Homologues to *H. somnus* 129PT	762	36.3%
Homologues to *E. coli* K12	540	25.8%

### Analysis of metabolism

**Figure 3:**
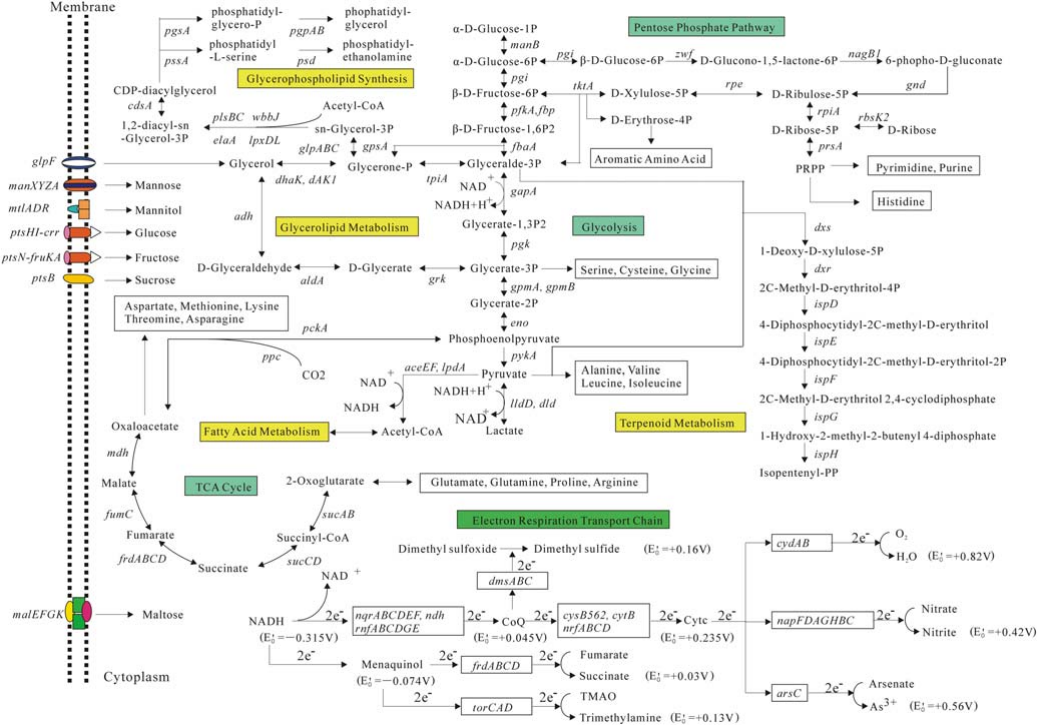
Overview of metabolic pathways in *A. pleuropneumoniae* JL03. Diagrammatic representation of carbon flow, electron flow, and biosynthesis of major metabolic intermediates and fatty acid are showed. Midpoint potentials (*E^'^_0_*) of some electron donors and acceptors of respiration chains are marked. Genes encoding crucial enzymes and functional proteins involved in the metabolic pathways are illustrated while the corresponding CDSs designated with APJL numbers are listed in Table S2.

A predicted set of genes encoding phosphotransferase systems (PTS) were identified in the genome of *A. pleuropneumoniae* JL03 supporting its utilization of various sugars, including mannose (*man*, APJL1410-1414), mannitol (*mtlADR*, APJL1663-1661), glucose (*ptsHI-crr*, APJL1336-1338), fructose (*ptsN-fruKA*, APJL0361-0359) and sucrose (*ptsB*, APJL1333) to generate energy *via* both fermentation and respiration (Figure 3). On the other hand, MalEFGK (APJL1249-1251, APJL1248) consist of an ABC (ATP-binding cassette) transport complex involved in maltose-specific transport system [19]. Concordantly, the CAP (*crp*, APJL2012)-cAMP (*cyaA*, APJL1072) system was annotated, which generally regulates the transcriptional rate of sugar utilization operons in multiple sugar utilization bacteria [20].

Besides fermentation, *A. pleuropneumoniae* performs both aerobic and anaerobic respirations and the latter is an important factor for pathogenesis (see Table S1). The electron transport chains in *A. pleuropneumoniae* might be branched and modular depending on its growth conditions (Figure 3). Cytochrome D ubiquinol oxidase encoded by *cydAB* (APJL0308, 0309) should be responsible for reducing the terminal electron acceptor oxygen to water in aerobic environments [21]. While, genes coding for various kinds of reductases specific for terminal electron acceptors of anaerobic respiration were also identified (Figure 3). Besides the arsenate reductase encoded by *arsC* (APJL1105), the *napFDAGHBC* (APJL1463-1457) operon encodes a periplasmic nitrate reductase system (NAP) highly homologous to that in *H. ducreyi* [22], which, as the sole nitrate reductase in *A. pleuropneumoniae*, should be essential to support anaerobic growth in the presence of nitrate [23]. Furthermore, albeit less favorable than nitrate, identification of *frdABCD* (APJL1556-1553) encoding a fumarate reductase and *dmsABC* (APJL1705-1707) encoding an anaerobic DMSO reductase in.JL03 inferred that this strain may be able to utilizing fumarate or dimethyl sulfoxide (DMSO) as electron acceptors as well (Figure 3) [4], [24].

Three global transcription regulators Hlyx (APJL0646), ArcA (APJL0049) and NarP (APJL0059) are encoded in all known genomes of *Pasteurellaceae,* including *A. pleuropneumoniae*. Under anaerobic conditions, these transcription factors may activate genes for anaerobic respiration while repress genes for aerobic respiration and fermentation [25].

Complete sets of genes coding for enzymes of glycolysis and gluconeogenesis, as well as non-oxidative pentose phosphate pathways were confirmed in strain JL03 (Figure 3). However, the tricarboxylic acid (TCA) cycle pathway in *A. pleuropneumoniae* was incomplete. Genes encoding three key enzymes of TCA cycle, *i.e.*, citrate synthase, aconitase and isocitrate dehydrogenase were not found in the genome. This pattern of metabolism was the same as species of genus of *Haemophilus*, e.g., *H. influenzae*, *H. ducreyi*, and *H. somnus* [11]. In addition, genes encoding malate synthase and isocitrate lyase, essential for glyoxylate pathway were also missing in JL03. Nevertheless, in JL03, the provision of C4 metabolites is unaffected and C5 metabolic intermediates should be offered by the non-oxidative synthesis process of the pentose phosphate pathway (Figure 3). In contrast to the bacteria in the genera *Actinobacillus* and *Haemophilus*, *P. multocida* and *M. succiniciproducens*, species of the genera *Pasteurella* and *Mannheimia* respectively, may perform their catabolism *via* an intact TCA cycle pathway [7].

The JL03 genome encodes almost all the enzymes involved in fatty acid metabolism, biosynthesis of glycerophospholipid, terpenoid, amino acid and purine/pyrimidine nucleotides (Figure 3). Interestingly, a specific operon *cysGHDNJI* (APJL1886-1881) encoding several proteins involved in assimilatory sulfate reduction was identified in JL03, and this operon is incomplete in the genome of *A. pleuropneumoniae* strain L20 (Figure 2B), and can not be found in the genomes of other members of the family *Pasteurellaceae* except for *M. succiniciproducens* MBEL55E. The assimilatory sulfate reduction has been extensively studied in *Escherichia coli* as a model for Gram-negative bacteria [26], [27], and the related genes, organized in a single operon *cysGHDNJI* in JL03, are dispersed into three operons in the genome of *E. coli* K12 MG1655 (U00096) [26]. The biological significance of the different genotypes and genomic organization deserves to further study.

JL03 needs nicotinamide adenine dinucleotide (NAD) for *in vitro* growth, but NAD is not required by *H. ducreyi* or *H. somnus* [11]. Concordantly, *H. ducreyi* 35000HP genome contains a duplication of the intact gene *nadV* (HD1447, 1455, 495aa) while *H. somnus* 129PT has one (HS0002, 465aa), which encodes the nicotinamide phosphoribosyltransferase (NAmPRTase) [11]. However, JL03 only bears a mutated *nadV*' CDS (APJL0638, 203aa) encoding merely a truncated domain. This is, for the first time, that genetic evidence was presented to support the previous notion that *A. pleuropneumoniae* serotype 3 belongs to the NAD-dependent biotype I category [28]. In addition, pathways involved in ubiquinone biosynthesis as well as riboflavin and vitamin B6 metabolism were also complete in JL03, as they are in *H. ducreyi* and *P. multocida*.

### Analysis of pathogenesis/virulence factors

Pathogenesis/virulence of *A. pleuropneumoniae* has been known to be related to many specific factors in addition to its metabolic features well adapted to *in vivo* growth and *in vitro* survive described above. The genomic characteristics of the specific pathogenesis/virulence factors are described in detail below:

#### APX exotoxins

Although the virulence of *A. pleuropneumoniae* is multifactorial, the major factor primarily responsible for the characteristic hemorrhagic lesions of the porcine contagious pleuropneumoniae are the pore-forming exotoxins belonging to the **r**epeat in **t**o**x**in (RTX) family [29], [30]. Widely distributed among Gram-negative bacteria, RTX toxins share structural and functional properties, including a characteristic nonapepetide glycine-rich repeat motif, a particular mode of secretion with a signal sequence at the C-terminus, post-translational activation, and cell toxicity via pore-forming mechanism [31]. RTX toxins in *A. pleuropneumoniae* are called Apx toxins (for *A. pleuropneumoniae* RTX toxins): the strongly hemolytic and cytotoxic ApxI, the weakly hemolytic and moderately cytotoxic ApxII, the nonhemolytic but strongly cytotoxic ApxIII, and the weakly hemolytic and cytotoxic ApxIV [1], [29]. Different serotypes secrete different sets of Apx toxins, causing variations in both of their hemolytic and cytotoxic activities [32]. Apx toxins are encoded by *apx* operons that usually consist of four contiguous genes arranged in the order of *apxCABD*. The *apxC* encodes a product that directs the cytoplasmic conversion by an acylation reaction of the structural toxin encoded by *apxA* to the active form, exported by a transporter encoded by *apxBD* [31]. The high degree of conservation of the RTX-B and RTX-D secretion proteins is reflected by the functional exchangeability of these proteins [33].

The absence of *apxICABD* operon in JL03 genome confirmed that it bore the moderate toxicity property of serotype 3, in contrast to serotypes 1, 5, 9, 10, and 11, all of which secrete ApxI [33]. Two tightly linked gene clusters, *apxIICAB*' (APJL0968-0966) and *apxIIICABD* (APJL1347-1344) [30], [34], were identified in the JL03 genome. The *apxII* operon is truncated in JL03, consisting of *apxIICA* but a partial *apxIIB*' without *apxIID*. It was evident that the secretion of ApxII may use the exporter encoded by *apxIBD* which are present in all serotypes except serotype 3, and that ApxII effect in serotype 3 is mainly cytoplasmic but barely hemolytic [29]. ApxIII has been known to be expressed and secreted by serotypes 2, 3, 4, 6, and 8 [29].

Remarkably, ApxIVA might be impaired in JL03 due to a Trp to nonsense mutation (tgG→tgA) in the coding region of this gene (APJL1015-1016). We further sequenced four independent isolates of serotype 3 and found that besides JL03, both S1421 and HB12 had the same TGA mutation. On the other hand, neither strain GDSB nor strain HV114 is mutated, bearing the prototype Trp codon (tgG). Concerning all of the genetic determinants of Apx encoded by JL03, it is worth mentioning that serotype 3 has very low virulence and secretes little ApxII, but normal amounts of ApxIII [29]. The absence of the most important operon *apxI* is likely to be an important factor leading to a decreased virulence of JL03.

#### Adherence

As previously reported, a 14-gene *flp* (fimbrial low-molecular-weight protein) operon (*flp1-flp2-tadV-rcpCAB-tadZABCDEFG*) has been found in the genera of *Haemophilus*, *Pasteurella*, *Pseudomonas, Yersinia, Caulobacter* and others, which is essential for Flp-pilus production, rough colony morphology, autoaggregation, and biofilm formation [34]. However, although JL03 possesses a series of genes encoding proteins responsible for bacterial adherence to host cells and biofilm formation (Table 5), the *flp* operon of it is truncated, composed of only 11 genes (APJL0549-0539), where the 5′-proximal *flp1*, *flp2* and *tadV* genes found in strain L20 were absent in JL03 (Figure 2C). In addition, the JL03 *rcpC* (APJL0549) is truncated with only a quarter of the C-terminal CDS maintained comparing to that of L20. The remaining JL03 *flp* operon genes were highly homologous to those from *H. ducreyi* 35000HP. For instance, the TadZABCDEFG CDSs have 66%, 88%, 71%, 75%, 70%, 54%, 52%, and 49% amino acid identity comparing to those of 35000HP, respectively [35]. However, RT-PCR experiments indicated that the *tadZABCDEFG* genes were not expressed in JL03 (data not shown) and it is likely due to the truncation of the promoter region of the JL03 *flp* operon, which was identified in strain L20. Because the Flp1 protein is the major structural component of Flp pili required for adherence-related phenotypes [34] and TadZABCDEFG is also known for tight adhesion [35], a truncated non-expression *flp* operon of strain JL03 should lead to failure of adherence.

**Table 5:** Genes encoding proteins with a role in adherence and secretion of strain JL03

CDS no.	Name	Function
APJL0201	*hofQ*	type II secretory protein
APJL0244	*secA*	preprotein translocase SecA subunit
APJL0539	*tadG*	Flp pilus assembly protein
APJL0540	*tadF*	tight adherence protein F
APJL0541	*tadE*	tight adherence protein E
APJL0542	*tadD*	Flp pilus assembly protein
APJL0543	*tadC*	Flp pilus assembly protein
APJL0544	*tadB*	Flp pilus assembly protein
APJL0545	*tadA*	tight adherence protein A
APJL0546	*tadZ*	Flp pilus assembly protein, ATPase
APJL0547	*rcpB*	rough colony protein B
APJL0548	*rcpA*	Flp pilus assembly protein, secretin
APJL0745	*secG*	preprotein translocase SecG subunit
APJL0889	*hopD*	leader peptidase
APJL0890	*hofC*	transport protein HofC homolog
APJL0891	*hofB*	pili/fimbriae biogenesis protein
APJL0892	*apfA*	possible prepilin peptidase dependent protein D
APJL1082	*yajC*	preprotein translocase YajC subunit
APJL1083	*secD*	protein-export membrane protein SecD
APJL1084	*secF*	protein-export membrane protein SecF
APJL1110	*dspB*	N-acetyl-beta-hexosaminidase
APJL1284	*pilF*	putative fimbrial biogenesis and twitching motility protein
APJL1456	*yidC*	preprotein translocase YidC subunit
APJL1535	*secB*	protein export protein
APJL1749	*secE*	preprotein translocase SecE subunit
APJL1815	*secY*	preprotein translocase SecY subunit
APJL1968	*pgaA*	biofilm PGA synthesis protein pgaA precursor
APJL1969	*pgaB*	biofilm PGA synthesis lipoprotein pgaB precursor
APJL1970	*pgaC*	biofilm PGA synthesis N-glycosyltransferase pgaC
APJL1971	*pgaD*	biofilm PGA synthesis protein
APJL2033	*tatA*	Sec-independent protein secretion pathway component
APJL2034	*tatB*	Sec-independent protein secretion pathway component
APJL2035	*tatC*	Sec-independent protein secretion pathway component

Polyglycolic acid (PGA), a linear polymer of N-acetyl-D-glucosamine residues in β-1,6 linkage, has been suggested to play a role in the intercellular adhesion and cellular detachment and dispersal in *A. actinomycetemcomitans* biofilm [36]. An operon consisted of *pgaABCD* genes (APJL1968-1971) encoding hexosamine-containing extracellular polysaccharide adhesin biosynthesis enzymes and another gene *dspB* (APJL1110) encoding N-acetyl-β-hexosaminidase were identified in the JL03 genome. The presence of these genes is consistent with the hypothesis that biofilm formation may be relevant to the colonization, pathogenesis and transmission of *A. pleuropneumoniae* [36].

#### Capsular polysaccharides (CPS)

Bacterial polysaccharides are extremely diverse and occur in multiple forms, with substantial variations within a species. They include CPS, exopolysaccharides (EPS) and O-antigens [37]. CPS is required for virulence of bacteria and variation in CPS content may contribute to the differences in virulence among *A. pleuropneumoniae* isolates [38]. A specific genomic island-like fragment, approximately 8.6 kb, encoding genes involved in CPS biosynthesis and export was identified in JL03 (Table 6). BLASTn searches revealed that the CPS biosynthetic enzymes encoded by the *cps3ABCD* (APJL1614-1611) operon were serotype 3-specific in strain JL03 (Figure 2D). The putative proteins encoded by *cps3A* and *cps3D* both have CDP-glycerol glycerophosphotransferase motifs. Cps3B contained a cytidylyltransferase motif. This is a key regulatory enzyme for phosphatidylcholine biosynthesis. The putative Cps3D was 57% similar to the TagF protein of *Campylobacter jejuni* involved in teichoic acid biosynthesis. The proteins encoded by the genes of the *cps* operon showed low homology with those encoded by genes of different *A. pleuropneumoniae* serotypes [39].

**Table 6:** Genes encoding proteins with a role in capsular polysaccharide biosynthesis of strain JL03

CDS no.	Name	Putative function
APJL1611	*cps3D*	teichoic acid biosynthesis protein
APJL1612	*cps3C*	Cps7C
APJL1613	*cps3B*	glycerol-3-phosphate cytidylyltransferase
APJL1614	*cps3A*	Cps2A
APJL1615	*cpxD*	capsule biosynthetic locus
APJL1616	*cpxC*	capsule polysaccharide export transport system permease protein
APJL1617	*cpxB*	capsule polysaccharide export transport system permease protein
APJL1618	*cpxA*	capsule polysaccharide export transport system ATP-binding protein
APJL1727	*lipA*	capsule polysaccharide modification protein
APJL1728	*phyB*	capsule biosynthetic locus

Upstream of *cps3A*, transcribed in the opposite orientation, there is another operon of four genes *cpxDCBA* (APJL1615-1618) encoding proteins involved in the export of CPS. These genes showed a high degree of homology to the group II capsule export genes *hexDCBA* in *P. multocida* strain Pm70 and *bexDCBA* in *H. influenzae* type b strain [23], [39]. Surprisingly, nucleotide sequences of the *cps* genes had no sequence similarity or seemingly no DNA rearrangement between JL03 of serotype 3 and L20 of serotype 5b. On the other hand, the *cps* genes seemed to be identical between L20 of serotype 5b and 4074 of serotype 1. These data suggested that the serotype diversity of *A. pleuropneumoniae* might be associated with CPS biosynthesis, but not with CPS export.

#### Lipopolysaccharide (LPS) biosynthesis

LPSs of Gram-negative bacteria are essential structural components of the cell membrane and are considered to be virulence determinants [40]. These substances are complex molecules composed of three well-defined biochemical moieties: the lipid A; the core oligosaccharide, which contains 3-deoxy-D-manno-oct-2-ulosonic acid (KDO) and is essential for optimal adhesion of *A. pleuropneumoniae* to the host [3]; and the O-antigen, a polysaccharide consisting of repeating sugar units [41].

We identified all the genes encoding enzymes for LPS biosynthesis in the JL03 genome (Table 7). Majority of the genes encoding enzymes for lipid A and KDO core biosynthesis were highly conserved among different species within the family *Pasteurellaceae* [11], [18]. These CDSs are scattered throughout the JL03 chromosome, like most Gram-negative prokaryotes, such as *E. coli*, *Neisseria meningitidis*, *Yersinia pestis*, *P. aeruginosa* and *Fusobacterium nucleatum*.

**Table 7:** Genes encoding enzymes with a role in lipopolysaccharide metabolism of strain JL03 and orthologs present in genomes of *A. pleuropneumoniae* L20 and 4074

CDS no.	Name	Putative function	L20	4074
APJL0008	*lpxB*	lipid-A-disaccharide synthase	++*	++
APJL0025	*lpxC*	UDP-3-O-acyl-GlcNAc deacetylase	++	++
APJL0051	-	3-deoxy-D-manno-octulosonate 8-phosphate phosphatase	++	++
APJL0085	*kdsB*	3-deoxy-manno-octulosonate cytidylyltransferase	++	++
APJL0173	*lpxM*	lipid A acyltransferase	++	++
APJL0423	*waaE*	ADP-heptose synthase	++	++
APJL0433	*lpxD*	UDP-3-O-(3-hydroxymyristoyl) glucosamine N-acyltransferase	++	++
APJL0446	*waaQ*	heptosyltransferase	++	++
APJL0641	*galU*	UTP-glucose-1-phosphate uridylyltransferase	++	++
APJL0804	*lpcA*	phosphoheptose isomerase	++	++
APJL0855	-	phosphatase	++	++
APJL0912	*lpxL*	lipid A acyltransferase	++	++
APJL0999	-	D-glycero-D-manno-heptosyltransferase	++	++
APJL1000	*lbgA*	lipooligosaccharide galactosyltransferase I	++	++
APJL1043	*lsgF*	putative UDP-galactose–lipooligosaccharide galactosyltransferase	++	++
APJL1044	*lsgE*	putative lipooligosaccharide galactosyltransferase	++	++
APJL1046	*lsgD*	glycosyltransferase involved in LPS biosynthesis	++	++
APJL1151	*waaA*	3-deoxy-D-manno-octulosonic-acid transferase	++	++
APJL1290	*lpxK*	tetraacyldisaccharide 4′ kinase	++	++
APJL1314	*galE*	UDP-glucose-4-epimerase	++	++
APJL1382	-	phosphoheptose isomerase	++	++
APJL1427	*rfaC*	lipopolysaccharide heptosyltransferase-1	++	++
APJL1428	*rfaF*	ADP-heptose LPS heptosyltransferase	++	++
APJL1485	*wzz*	Wzz homolog	−	−
APJL1486	*rfaG*	lipopolysaccharide biosynthesis glycosyltransferase	−	−
APJL1487	-	VI polysaccharide biosynthesis protein	−	−
APJL1488	-	glycosyltransferase	−	−
APJL1489	-	glycosyltransferase	−	−
APJL1490	*wzy*	oligosaccharide repeat unit polymerase	−	−
APJL1491	*wzx*	flippase Wzx	−	−
APJL1492	*glf*	UDP-galactopyranose mutase	+	+
APJL1493	-	putative sugar transferase	+	+
APJL1494	-	acyltransferase 3	−	−
APJL1496	-	polysacchride biosynthesis protein	−	−
APJL1497	*rmlB*	dTDP-glucose 4,6-dehydratase	++	++
APJL1576	*wzxE*	lipopolysaccharide biosynthesis protein	++	++
APJL1577	*rffA*	lipopolysaccharide biosynthesis protein rffA	++	++
APJL1578	*rffC*	lipopolysaccharide biosynthesis protein rffC	++	++
APJL1582	*wecA*	undecaprenyl-phosphatealpha-N-acetylgluco- saminyltransferase	++	++
APJL1742	*gmhD*	ADP-L-glycero-D-manno-heptose-6-epimerase	++	++
APJL1844	*lpxH*	UDP-2,3 diacylglucosamine hydrolase	++	++
APJL1938	*lgt*	prolipoprotein diacylglyceryl transferase	++	++
APJL2091	*kdsA*	2-dehydro-3-deoxyphosphooctonate aldolase	++	++

* ++ represents identity>80%; + represents 50%>identity>30%; − represents no homologous protein

A cluster of genes coding for enzymes that catalyze the biosynthesis of O-antigen was identified in JL03 ranging from *wzz* (APJL1485) to *rmlB* (APJL1497), of which, only the dTDP-glucose 4,6-dehydratase RmlB was conserved across a wide range of species. These CDSs could be divided into three groups [37]: nucleotide sugar biosynthesis related (APJL1496 and 1497); glycosyltransferases (APJL1486-1489 and 1493) involved in sugar transfer; and oligosaccharide repeat unit processing related, *wzz*, *wzy* and *wzx* (APJL1490 and 1491).

**Figure 4:**
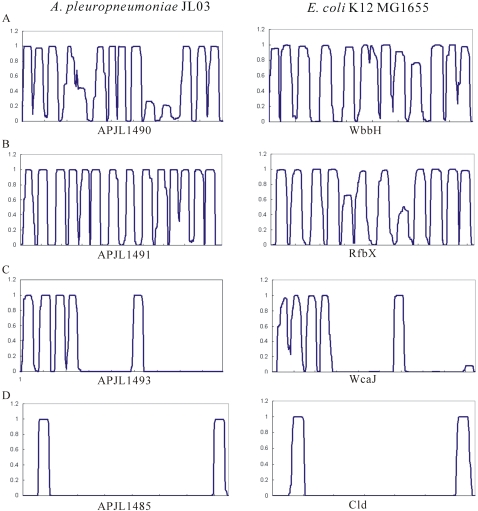
Schematic illustration of functional assignment to genes coding for enzymes for O-antigen biosynthesis based on predicting the topology of transmembrane proteins. All the amino acid sequences are from the genomes of *A. pleuropneumoniae* JL03 and *E. coli* K12 MG1655. CDSs designated with numbers and corresponding annotations are listed below: A. *wzy* (APJL1490) (*wbbH*), encoding oligosaccharide repeat unit polymerase; B. *wzx* (APJL1491) (*rfbX*), encoding O-antigen flippase; C. *wcaJ* (APJL1493), encoding glycosyltransferase; D. *wzz* (APJL1485) (*cld*), encoding an O-antigen chain length determining protein.

A bacterial sugar transferase (436aa) encoded by APJL1493 shares 55% identity with Orf9 (400aa) found in *A. actinomycetemcomitans* [42], but only 34% identity with a sugar transferase (472aa) encoded by APL1471 of strain L20. Two proteins (encoded by APJL1487 and 1488) among the four closely linked glycosyl transferases contain a *Glycos_transf_1* domain (PF00534) in their C-termini and a *Glycos_transf_2* domain (PF00535) in their N-termini, respectively, both unique among *Pasteurellaceae* species. Gene *wzz* encodes a protein (370aa) bearing 45% identity with the O-antigen chain length determining protein (MHA1853, 375aa) found in *M. haemolytica* [18]. Although there are much sequence variabilities among the O-antigen-processing enzymes in different Gram-negative bacteria, structural conservation and stability of membrane spanning regions still indicate that they should perform similar function predicted by the numbers and loci of relevant transmembrane helices (TMHs) (Figure 4).

The G+C content of the gene cluster coding for enzymes responsible for O-antigen chain biosynthesis was much lower (31%) than that of the JL03 chromosome (41%). On the other hand, genomic comparison with strains L20 or 4074 revealed that these CDSs were more variable than those for the synthesis of LPS lipid A and core oligosaccharide between serotypes (Table 7).

As defined by the sequences of these serotype-specific CDSs, serotype 3 should be able to be distinguished from serotype 5b and 1 as another group (Figure 2E). Therefore, comparison of the O-antigen regions, which were analogous with that of the *cps* operons, could also be used as one of the markers in classifying serotypes of *A. pleuropneumoniae*.

#### Virulence related enzymes

Various enzymes, such as urease and proteases, are known to play important roles in the disease process of *A. pleuropneumoniae*. Quite a few respiratory tract pathogens produce urease, which catalyzes the hydrolysis of urea to produce ammonia and carbon dioxide [43]. As previously reported [44], the gene cluster (*ureABC*, APJL1651-1649) identified in the genome of JL03 encodes the structural subunits of urease while the closely linked genes *ureEFGH* (APJL1647-1644) encode the accessory subunits. All of these genes are orthologues of those in the *ure* operon of *H. influenzae*. Furthermore, a 6-gene cluster, upstream of the *ure* operon and transcribed in the same direction, was also identified. Five of the aforementioned six genes formed a *cbi* operon (APJL1657-1652) encoding a putative nickel and cobalt periplasmic permease system, which may affect the total urease activity in *A. pleuropneumoniae* [44].

The *pepN* (APJL1358) encoding aminopeptidase N was identified in JL03. It was characterized on the basis of a zinc binding motif (aa 294-303) found in metalloproteases. Expression of this protease was observed in lung tissue of pigs that had died from porcine pleuropneumonia [45].

Genes encoding the enzymes required for anaerobic respiration, *i.e.*, periplasmic nitrate reductase and DMSO reductase were described above. These enzymes are probably accessory virulence factors in *A. pleuropneumoniae* pathogenesis [4].

#### Iron acquisition and utilization

**Figure 5:**
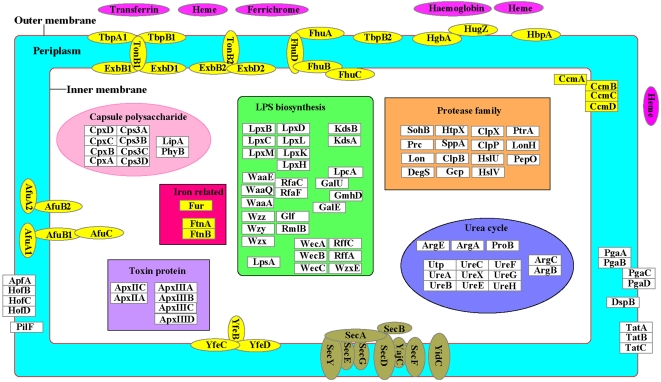
A schematic diagram of virulence factors located in virtual cell environment of *A. pleuropneumoniae* JL03. CDSs corresponding to the illustrated proteins with designated APJL numbers are listed in Table 5 (adherence and secretion relevant genes), Table 6 (capsule polysaccharide relevant genes), Table 7 (lipopolysaccharide relevant genes), Table S3 (iron relevant genes) and S4 (the rest portion relevant genes). Proteins involved in iron uptake, transport and regulation are colored in yellow.

Iron is essential for bacterial growth and acts as an environmental signal that regulates the expression of many virulence factors [3]. Mammals have evolved a mechanism to reduce the availability of iron to potential bacterial pathogens by using of very-high-affinity iron-chelating molecules, while host-adapted pathogens have accordingly evolved means to use these iron-bearing molecules as an iron source [46]. It is known that *A. pleuropneumoniae* can use porcine transferrin, hemoglobin and ferrichrome [3], [46]. Approximately 2.6% (55 genes) of the JL03 genome are involved in iron uptake with additional 5 related pseudogenes likely impaired by mutations. Comparing with the genomes of other *Pasteurellaceae* members, large proportion of genes involved in iron metabolism seems common at least within the family (Table S3). These iron metabolism related proteins are highly conserved between JL03 and L20 except for TbpB1 (APJL1598) and FhuA (APJL2066). An analogous cell model of some iron-related protein complexes and other virulence factors is illustrated in Figure 5.

The TonB system plays a key role in iron acquisition by many Gram-negative bacteria. Two functional TonB systems were reported in detail for *P. aeruginosa* [47] and *A. pleuropneumoniae* [46]. Two sets of closely linked genes encoding the TonB1 and TonB2 systems, namely *tonB1*(246aa)-*exbB1*-*exbD1* (APJL1601-1599) and *exbB2*-*exbD2*-*tonB2* (244aa) (APJL0078-0076), were identified in the JL03 genome. The identity between *tonb1/tonb2* was only 15%, suggesting two structurally independent TonB systems for iron uptake. Transferrin-binding protein (Tbp), a kind of iron receptors, has been found in many species of the families *Pasteurellaceae* and *Neisseriaceae* [3]. Two pairs of *tbp* genes were found in the JL03 genome, *tbpB1*-*tbpA1* (APJL1598-1597) and *tbpB2*-*tbpA2*′ (partial)-*tbpA2*′ (partial) (APJL0250-0252). Moreover, the nucleotide sequence identity between the two sets of genes was 54.4%, indicating that they were likely to be duplicated copies. The *tbpB1*-*tbpA1* operon was located immediately downstream of the *tonB1*. It is unclear whether these two sets of Tbps are functional. If they are both functional, it would also be interesting to learn the corresponding TonB system relevant to each set of Tbps.

The *fhu* operon encodes CDSs homologous to proteins involved in the uptake of hydroxamate siderophore across the outer membrane of several bacteria [3]. The *fhuCDBA* (APJL2063-2066) genes encode four proteins with 28.5 kDa, 35.8 kDa, 69.4 kDa and 77.1 kDa in sequential order, in agreement with previous studies [48]. Orthologs of *ccmABCDEF* (APJL1390-1385) were found in *H. ducreyi* and are involved in post-translational attachment of heme and catalyze the reduction of disulfide bonds in the cytochrome c apoprotein [22], [49]. Both *ccmE* and *ccmF* have multiple TMHs and probable signal peptide in their N-termini. A number of genes that encode putative iron-binding receptors were found in JL03 in addition to the *tbp* genes, such as two *hbpA* genes (APJL0866 and 2060) encoding the heme-binding protein A and an outer membrane iron-receptor protein (99 kDa, APJL1922). JL03 also has the *hgbA* gene (APJL1065) encoding a haemoglobin-binding protein located at the outer membrane, which is regulated by a highly conserved gene *fur* (APJL1231) encoding ferric uptake regulator Fur [50], [51]. Upstream of *hgbA*, there is a CDS encoding a potential haemin-binding protein homologous to the HugZ in *Plesiomonas shigelloides* [50].

Thus, a remarkably large number of genes encoding putative iron uptake proteins were found in the genome of JL03. *A. pleuropneumoniae* appears well-equipped to overcome iron shortages during infection.

In summary, we have sequenced the complete genome of *A. pleuropneumoniae* strain JL03, a Chinese field isolate of serotype 3 and annotated the genome in comparison against other members of the *Pasteurellaceae* family. A complicated metabolic network with various kinds of oxidation-reduction enzymes for catabolism and anabolism was comprehensively illustrated at the genomics level for this genus, and, for the first time, genomic discoveries were made to account for assimilatory sulfate reduction (intact operon *cysGHDNJI*) and NAD-dependent biotype I character (truncated *nadV*) of the strain (Figure 3). Meanwhile, we identified a series of genes encoding proteins of Apx toxins, adhesins, iron-uptake systems as well as enzymes for the biosynthesis of CPS and LPS, which underlined the genetic basis related to the pathogenesis/virulence of *A. pleuropneumoniae*. Furthermore, comparing to the genomes of strains L20 (serotype 5b) and 4074 (serotype 1) of *A. pleuropneumoniae*, probable strain (serotype)-specific genomic islands and genome reductions were identified in JL03. These data should provide a foundation for future research into the mechanisms of virulence and serotype diversity of *A. pleuropneumoniae*.

## Materials and Methods

### Bacterial strain

The *A. pleuropneumoniae* strain JL03 used for genomic sequencing was isolated from the lung of a pig from a Chinese commercial pig farm in 2003 [52] This isolate was identified as serotype 3 [53], [54] and was deposited in China Center for Type Culture Collection (CCTCC, Wuhan) available upon request. It grows well at 37°C on Tryptic Soy Agar or in Tryptic Soy Broth, supplemented with 10 mg/ml nicotinamide adenine dinucleotide (NAD) and 5% bovine serum.

### Genomic sequencing, assembly and analysis

A whole genome shotgun strategy was adopted. Two genomic DNA libraries of JL03 with 1.5–4 kb or 6–8 kb insertion fragments were constructed in pUC18 or pSmart-LC respectively. Total of 13,440 clones (10,080 from the 1.5–4 kb pUC18 clones and 3,360 from the 6–8 kb pSmart-LC clones) were sequenced from both ends by ABI 3700 DNA analyzer, and altogether, 25,650 sequencing reads (Phred value>Q20, of which 760 bp was the confirmed mean length of reads) gave an 8.6-fold coverage of the genome. Employing Phred [55] and the Staden software package [56], 170 contigs were assembled. Sequence and physical gaps of the unfinished genome were filled by primer walking with 226 effective PCRs. The final closure was confirmed by sequencing the PCR amplified corresponding contig-connecting fragments using the JL03 genomic DNA as the template. The finished complete genomic sequence was analyzed by conventional genomic annotation methods, which was described in detail in the Text S1 as *Materials and Methods*.

## Supporting Information

Table S1 Orthologs comparison of genes involved in respiration, central metabolism and corresponding regulation
(0.32 MB DOC)

Table S2 Crucial enzymes and functional proteins involved in metabolic pathways of *A. pleuropneumoniae* strain JL03
(0.13 MB DOC)

Table S3 Genes encoding proteins involved in iron metabolism of *A. pleuropneumoniae* JL03 compared with the homologous proteins from three representative genomes within *Pasteurellaceae*
(0.19 MB DOC)

Table S4 Genes encoding proteins associated with virulence factors (apx toxins, proteases, and urease) in *A. pleuropneumoniae* JL03
(0.06 MB DOC)

Text S1 Supplementary Materials and Methods
(0.04 MB DOC)
