# Supplementary material for: Genome Biology of Actinobacillus pleuropneumoniae JL03, an Isolate of Serotype 3 Prevalent in China
Source: PLoS One. 2008 Jan 16;3(1):e1450. doi: 10.1371/journal.pone.0001450 (PMC2175527; doi:10.1371/journal.pone.0001450)
Supplement: Table S1 — Orthologs comparison of genes involved in respiration, central metabolism and corresponding regulation (0.32 MB DOC) [file pone.0001450.s001.doc]

**Table S1**. Orthologs comparison of genes involved in respiration, central metabolism and corresponding regulation

| Gene name | Organisms* | | | | | | Gene function |
| --- | --- | --- | --- | --- | --- | --- | --- |
|  | A.P. | P.M. | M.S. | E.C. | P.A. | F.N |  |
| Aerobic | | | | | | | |
| *cydA* | APJL0308 | PM0974 | MS0715 | b0733 | - | - | cytochrome D ubiquinol oxidase, subunit I |
| *cydB* | APJL0309 | PM0973 | MS0716 | b0734 | - | - | cytochrome D ubiquinol oxidase, subunit II |
| *napB* | APJL1458 | PM1597 | MS2278 | b2203 | PA1173 | **-** | nitrate reductase cytochrome c-type subunit |
| *napA* | APJL1461 | PM1594 | MS2281 | b2206 | PA1174 | **-** | periplasmic nitrate reductase |
| *appC* | - | - | - | b0978 | PA3930 | - | cytochrome bd-II oxidase, subunit I |
| *appB* | - | - | - | b0979 | PA3929 | - | cytochrome bd-II oxidase, subunit II |
| *cyoE* | - | - | - | b0428 | PA1321 | - | protoheme IX farnesyltransferase |
| *cyoD* | - | - | - | b0429 | PA1320 | - | cytochrome o ubiquinol oxidase subunit IV |
| *cyoC* | - | - | - | b0430 | PA1319 | - | cytochrome o ubiquinol oxidase subunit III |
| *cyoB* | - | - | - | b0431 | PA1318 | - | cytochrome o ubiquinol oxidase subunit I |
| *cyoA* | - | - | - | b0432 | PA1317 | - | cytochrome o ubiquinol oxidase subunit II |
| Anaerobic | | | | | | | |
| *nrfA* | APJL0100 | PM0023 | MS1819 | b4070 | - | - | nitrate reductase, cytochrome c552 |
| *nrfB* | APJL0101 | PM0024 | MS1818 | b4071 | - | - | nitrate reductase, cytochrome-c protein |
| *nrfC* | APJL0102 | PM0025 | MS1817 | b4072 | - | - | nitrate reductase, Fe-S protein |
| *nrfD* | APJL0103 | PM0026 | MS1816 | b4073 | - | - | nitrate reductase, transmembrane protein |
| *glpA* | APJL0398 | PM1442 | MS1993 | b2241 | - | - | anaerobic glycerol-3-phosphate dehydrogenase,subunit A |
| *glpB* | APJL0399 | PM1441 | MS1994 | b2242 | - | - | anaerobic glycerol-3-phosphate dehydrogenase,subunit B |
| *glpC* | APJL0400 | PM1440 | MS1995 | b2243 | - | - | anaerobic glycerol-3-phosphate dehydrogenase,subunit C |
| *fdhD* | APJL0903 | PM0410 | MS0893 | b3895 | PA5180 | - | formate dehydrogenase formation protein |
| *bisC1* | APJL0904 | PM0409 | MS0892 | - | - | - | formate dehydrogenase-N, alpha subunit |
| *bisC2, fdnG* | APJL0905 | PM0408 | MS0891 | b1474 | - | - | formate dehydrogenase-N, alpha subunit |
| *hybA, fdnI* | APJL0906 | PM0407 | MS1029 | b3893 | PA4811 | - | formate dehydrogenase-O, Fe-S subunit |
| *fdnI* | APJL0907 | PM0406 | MS0889 | b1476 | PA4810 | - | formate dehydrogenase, gamma subunit |
| *fdhE* | APJL0908 | PM0405 | MS0843 | b3891 | PA4809 | - | formate dehydrogenase formation protein |
| *nrfG* | APJL1067 | PM0030 | MS1811 | b4076 | PA1483 | - | formate-dependent nitrite reductase complex |
| *ccmH* | APJL1068 | PM0029 | MS1812 | b4075 | - | - | cytochrome c-type protein |
| *nfrE* | APJL1070 | PM0027 | MS1815 | b4074 | - | - | cytochrome c-type biogenesis protein |
| *frdD* | APJL1553 | PM0198 | MS1655 | b4151 | - | - | fumarate reductase |
| *frdC* | APJL1554 | PM0199 | MS1654 | b4152 | - | - | fumarate reductase |
| *frdB* | APJL1555 | PM0200 | MS1653 | b4153 | - | - | fumarate reductase iron-sulfur protein |
| *frdA* | APJL1556 | PM0201 | MS1652 | b4154 | - | FN0009 | fumarate reductase flavoprotein subunit |
| *dmsA* | APJL1705 | PM1754 | - | b0894 | - | - | anaerobic dimethyl sulfoxide reductase chain A precursor |
| *dmsB* | APJL1706 | PM1755 | - | b0895 | - | - | anaerobic dimethyl sulfoxide reductase chain B |
| *dmsC* | APJL1707 | PM1756 | MS2336 | b1590 | - | - | anaerobic dimethyl sulfoxide reductase chain C |
| *narG* | - | - | - | b1224 | PA3875 | - | nitrate reductase 1, alpha subunit |
| *narH* | - | - | - | b1225 | - | - | nitrate reductase 1, beta (Fe-S) subunit |
| *narJ* | - | - | - | b1226 | PA3873 | - | nitrate reductase |
| *narI* | - | - | - | b1227 | - | - | nitrate reductase |
| ATP-proton motive force interconversion | | | | | | | |
| atpC | APJL1678 | PM1495 | MS2345 | b3731 | PA5553 | - | ATP synthase epsilon chain |
| *atpD* | APJL1679 | PM1494 | MS2346 | b3732 | PA5554 | FN0358 | ATP synthase beta chain |
| *atpG* | APJL1680 | PM1493 | MS2347 | b3733 | PA5555 | FN0359 | ATP synthase gamma chain |
| *atpA* | APJL1681 | PM1492 | MS2348 | b3734 | PA5556 | FN0360 | ATP synthase alpha chain |
| *atpH* | APJL1682 | PM1491 | MS2349 | b3735 | PA5557 | - | ATP synthase delta chain |
| *atpF* | APJL1683 | PM1490 | MS2350 | b3736 | PA5558 | - | ATP synthase B chain |
| *atpE* | APJL1684 | PM1489 | MS2351 | - | PA5559 | - | ATP synthase C chain |
| *atpB* | APJL1685 | PM1488 | MS2352 | b3738 | PA5560 | FN0364 | ATP synthase A chain |
| *atpI* | APJL1686 | - | - | - |  | - | ATP synthase protein I |
| *atpE* | - | - | - | b3737 | - | - | - |
| Electron transport | | | | | | | |
| *fdx1* | APJL0058 | PM1858 | MS0247 | b2562 | PA0362 | - | ferredoxin-like protein |
| *trxB* | APJL0084 | PM0573 | MS0951 | b0888 | PA2616 | FN1984 | thioredoxin reductase |
| *nqrA* | APJL0151 | PM1328 | MS0309 | - |  | - | NADH-ubiquinone oxidoreductase subunit A |
| *nqrB* | APJL0152 | PM1329 | MS0308 | - | PA2998 | - | NADH-ubiquinone oxidoreductase subunit B |
| *nqrC* | APJL0153 | PM1330 | MS0307 | - | PA2997 | - | NADH-ubiquinone oxidoreductase subunit C |
| *nqrD* | APJL0154 | PM1331 | MS0306 | - | PA2996 | - | NADH-ubiquinone oxidoreductase subunit D |
| *nqrE* | APJL0155 | PM1332 | MS0305 | - | PA2995 | - | NADH ubiquinone oxidoreductase subunit 5 |
| *rnfA* | APJL0166 | PM0387 | MS0922 | b1267 | PA3199 | FN1592 | NADH-quinone oxidoreductase |
| *rnfB* | APJL0167 | PM0386 | MS0921 | b1628 | PA3490 | FN1591 | ferredoxin II, iron sulfur protein |
| *rnfC* | APJL0168 | PM0385 | MS0920 | b1629 | PA3491 | FN1596 | iron-sulfur binding NADH dehydrogenase |
| *rnfD* | APJL0170 | PM0384 | MS0919 | b1630 | PA3492 | FN1595 | NADH-quinone reductase |
| *rnfG* | APJL0171 | PM0383 | MS0918 | b1631 | PA3493 | - | electron transport complex protein |
| *rnfE* | APJL0172 | PM0382 | MS0917 | b1632 | PA3494 | FN1593 | Na-translocating NADH-quinone reductase |
| *ykgF* | APJL0472 | PM1854 | MS0751 | b0307 | - | FN1540 | iron-sulfur electron transport protein |
| *pntB* | APJL0849 | PM0752 | MS1223 | b1602 | PA0196 | - | NAD(P) transhydrogenase, beta subunit |
| *pntA* | APJL0850 | PM0753 | MS1224 | b1603 | PA0195 | - | NAD/NADP transhydrogenase alpha subunit |
| *fdx2* | APJL0934 | PM0323 | MS1720 | b2525 | PA3809 | - | ferredoxin |
| *trxA* | APJL1094 | PM0994 | MS1626 | b3781 | PA5240 | FN0093 | thioredoxin |
| *nfnB* | APJL1214 | PM0733 | MS1349 | - | PA5190 | - | NAD(P)H nitroreductase |
| *fldA* | APJL1232 | PM0353 | MS0860 | b0684 | - | FN0724 | flavodoxin A |
| *lpd, gor* | APJL1255 | PM1235 | MS1985 | b3500 | PA2025 | FN0820 | glutathione oxidoreductase |
| *ccmF* | APJL1385 | PM0010 | MS0606 | b2196 | PA1480 | - | cytochrome c-type biogenesis protein |
| *ccmE* | APJL1386 | PM0009 | MS0605 | b2197 | PA1479 | - | cytochrome c-type biogenesis protein |
| *napC* | APJL1457 | PM1598 | MS2277 | b2202 | PA1172 | - | cytochrome c subunit |
| *napH* | APJL1459 | PM1596 | MS2279 | b2204 | - | - | polyferredoxin |
| *napG* | APJL1460 | PM1595 | MS2280 | b2205 | - | - | ferredoxin 2 |
| *napF* | APJL1463 | PM1592 | MS2283 | b2208 | PA1176 | - | ferredoxin-type protein |
| *ccmH1* | APJL1541 | PM0012 | MS0608 | - | - | - | cytochrome c-type biogenesis protein |
| *ccmH2* | APJL1542 | PM0013 | MS0609 | b2194 | PA1482 | - | cytochrome c-type biogenesis protein |
| *torD* | APJL1833 | PM1794 | - | b0998 | - | - | trimethylamine-n-oxide oxidoreductase |
| *torA* | APJL1834 | PM1793 | MS0588 | b0997 | - | - | trimethylamine-n-oxide reductase precursor |
| *torC* | APJL1835 | PM1792 | MS0587 | b0996 | - | - | cytochrome c-type protein |
|  | - | PM0446 | - | - | - | - | cytochrome C-553 homolog |
| *resA* | - | PM0447 | - | - | - | - | cytochrome c-type biogenesis protein |
| *ttrA* | - | PM0721 | - | - | - | - | tetrathionate reducatase |
| *ttrC* | - | PM0722 | - | - | - | - | tetrathionate reducatase |
| *ttrB* | - | PM0723 | - | b1467 | PA3874 | - | tetrathionate reducatase |
| *trx* | - | PM1705 | - | - | - | - | thioredoxin |
| *nuoA* | - | - | - | b2288 | PA2637 | - | NADH dehydrogenase subunit A |
| *nuoB* | - | - | - | b2287 | PA2638 | - | NADH dehydrogenase subunit B |
| *nuoc, nuoD* | - | - | - | b2286 | PA2639 | - | NADH:ubiquinone oxidoreductase, chain C,D |
| *nuoE* | - | - | - | b2285 | PA2640 | - | NADH dehydrogenase subunit E |
| *nuoF* | - | - | - | b2284 | PA2641 | - | NADH:ubiquinone oxidoreductase, chain F |
| *nuoG* | - | - | - | b2283 | PA2642 | - | NADH dehydrogenase subunit G |
| *nuoH* | - | - | - | b2282 | PA2643 | - | NADH dehydrogenase subunit H |
| *nuoI* | - | - | - | b2281 | PA2644 | - | NADH dehydrogenase subunit I |
| *nuoJ* | - | - | - | b2280 | PA2645 | - | NADH dehydrogenase subunit J |
| *nuoK* | - | - | - | b2279 | PA2646 | - | NADH dehydrogenase subunit K |
| *nuoL* | - | - | - | b2278 | PA2647 | - | NADH dehydrogenase subunit L |
| *nuoM* | - | - | - | b2277 | PA2648 | - | NADH dehydrogenase subunit M |
| *nuoN* | - | - | - | b2276 | PA2649 | - | NADH dehydrogenase subunit N |
| *sdhC* | - | - | - | b0721 | PA1581 | - | succinate dehydrogenase cytochrome b556 large membrane subunit |
| *sdhD* | - | - | - | b0722 | PA1582 | - | succinate dehydrogenase cytochrome b556 small membrane subunit |
| *sdhA* | - | - | - | b0723 | PA1583 | - | succinate dehydrogenase flavoprotein subunit |
| *sdhB* | - | - | - | b0724 | PA1584 | - | succinate dehydrogenase, FeS subunit |
| Fermentation | | | | | | | |
| *ydfG* | APJL0495 | PM0579 | MS1568 | b1539 | PA4907 | FN1433 | NADP-dependent dehydrogenase |
| *adh2* | APJL1029 | PM1453 | MS2190 | b1241 | - | FN0084 | acetaldehyde dehydrogenase [acetylating] |
| *act* | APJL1053 | PM0077 | MS0403 | b0902 | - | FN0261 | pyruvate formate-lyase activating enzyme |
| *pflB* | APJL1054 | PM0075 | MS0401 | b0903 | - | FN0262 | formate acetyltransferase |
| *srlD* | - | PM1968 | - | b2705 | - | - | sorbitol-6-phosphate 2-dehydrogenase |
| Glycolysis | | | | | | | |
| *pykA* | APJL0188 | PM0653 | MS1197 | b1854 | PA4329 | FN1765 | pyruvate kinase |
| *gpmB* | APJL0235 | PM0634 | MS1172 | b4395 | - | FN0808 | phosphoglycerate mutase |
| *fruK* | APJL0360 | PM1796 | MS2179 | b2168 | PA3561 | FN1440 | 1-phosphofructokinase |
| *gapdH* | APJL0460 | PM0924 | MS1739 | b1779 | - | FN0652 | glyceraldehyde 3-phosphate dehydrogenase |
| *pckA* | APJL0805 | PM1542 | MS2293 | b3403 | PA5192 | FN1120 | phosphoenolpyruvate carboxykinase |
| *gpmA* | APJL0840 | PM1506 | MS2321 | b0755 | - | FN0729 | phosphoglycerate mutase |
| *eno* | APJL1132 | PM1871 | MS0256 | b2779 | PA3635 | FN1764 | enolase |
| *pfkA* | APJL1143 | PM0069 | MS0377 | b3916 | - | FN0410 | 6-phosphofructokinase |
| *pgi* | APJL1156 | PM0416 | MS1181 | b4025 | PA4732 | - | glucose-6-phosphate isomerase |
| *fba* | APJL1261 | PM1861 | MS0244 | b2925 | - | - | fructose-bisphosphate aldolase |
| *pgk* | APJL1262 | PM1860 | MS0245 | b2926 | PA0552 | FN0654 | phosphoglycerate kinase |
| *fbp* | APJL1420 | PM0930 | MS1615 | b4232 | PA5110 | - | fructose-1,6-bisphosphatase |
| *gntK, glk* | APJL1697 | PM0792 | MS0957 | b4268 | PA2321 | - | gluconate kinase |
| *lldD* | APJL1891 | PM0288 | - | b3605 | PA4771 | - | L-lactate dehydrogenase |
| *tpiA* | APJL1972 | PM1311 | MS0324 | b3919 | PA4748 | FN1366 | triosephosphate isomerase |
| *rbsK* | APJL2085 | PM1849 | MS1233 | b3526 | PA2261 | - | sugar kinase |
| *acsA* | - | PM0692 | - | b4069 | PA0887 | - | acetyl-CoA synthetase |
| *fbaA* | - | PM1373 | - | b2096 | - | - | fructose-1,6-bisphosphate aldolase |
| *tpiA* | - | PM1640 | MS0379 | - | - | - | triosephosphate isomerase |
| *pfkB* | - | - | - | b1723 | - | - | 6-phosphofructokinase II |
| *dhnA* | - | - | - | b2097 | - | - | fructose-bisphosphate aldolase |
| *epd* | - | - | - | b2927 | PA0551 | - | D-erythrose 4-phosphate dehydrogenase |
| *pykF* | - | - | - | b1676 | - | - | pyruvate kinase |
| TCA cycle | | | | | | | |
| *ppc* | APJL0355 | PM0546 | MS1017 | b3956 | PA3687 | - | phosphoenolpyruvate carboxylase |
| *sucD* | APJL0478 | PM0281 | MS1351 | b0729 | PA1589 | - | succinyl-CoA synthetase alpha chain |
| *sucC* | APJL0479 | PM0280 | MS1352 | b0728 | PA1588 | - | succinyl-CoA synthetase beta chain |
| *sucB* | APJL0481 | PM0278 | MS1354 | b0727 | PA1586 | - | dihydrolipoamide succinyltransferase |
| *sucA* | APJL0482 | PM0277 | MS1355 | b0726 | PA1585 | - | 2-oxoglutarate dehydrogenase E1 component |
| *maeA,mdh* | APJL0515 | PM0002 | MS0390 | b2463 | PA5046 | - | malate oxidoreductase |
| *mdh* | APJL1309 | PM0550 | MS1266 | b3236 | - | - | malate dehydrogenase |
| *fumC* | APJL1792 | PM0823 | MS0760 | b1611 | PA0854 | - | fumarate hydratase |
| *acnB* | - | PM0204 | MS2369 | b0118 | PA1787 | - | aconitate hydrase B |
| *gltA* | - | PM0276 | MS2371 | b0720 | PA1580 | - | citrate synthase |
| *tdp,icd* | - | PM1606 | MS2370 | b1136 | PA2623 | - | isocitrate dehydrogenase |
| *acnA* | - | - | - | b1276 | PA1562 | - | aconitate hydratase |
| Pyruvate dehydrogenase | | | | | | | |
| *lpdA* | APJL0774 | PM0893 | MS1334 | b0116 | PA1587 | - | dihydrolipoamide dehydrogenase |
| *aceF* | APJL0775 | PM0894 | MS1335 | b0115 | PA5016 | - | dihydrolipoamide s-acetyltransferase |
| *aceE* | APJL0776 | PM0895 | MS1336 | b0114 | PA5015 | - | pyruvate dehydrogenase E1 component |
| Regulatory genes | | | | | | | |
| *arcA* | APJL0049 | PM0219 | MS1504 | b4401 | PA4983 | - | aerobic respiration control protein |
| *narP* | APJL0059 | PM1810 | MS0278 | b2193 | - | FN0189 | nitrate/nitrite response regulator protein |
| *narQ* | APJL0489 | PM1591 | MS2288 | b2469 | - | - | nitrate/nitrite sensor protein |
| *hlyX, fnr* | APJL0646 | PM0668 | MS1077 | b1334 | PA1544 | - | fnr-like transcriptional regulator protein |
| *arcB* | APJL0945 | PM0313 | MS1730 | b3210 | - | - | sensor histidine kinase |
| *phoR* | APJL1267 | PM0431 | - | b0400 | PA5361 | - | phosphate regulon sensor protein |
| *phoB* | APJL1268 | PM0432 | - | b0399 | PA5360 | - | phosphate regulon transcriptional regulatory protein |
| *crp* | APJL2012 | PM1157 | MS1934 | b3357 | PA0652 | - | catabolite gene activator |
| *torS* | - | - | - | b0993 | - | - | hybrid sensory histidine kinase |
| *torR* | - | - | - | b0995 | - | - | DNA-binding response regulator |

* The abbreviations of organisms are listed as follows: A.P., *A. pleuropneumoniae* JL03 (accession no. CP000687); P.M., *P. multocida* Pm70 (accession no. AE004439); M.S., *M. succiniciproducens* MBEL55E (accession no. AE016827); E.C., *E. coli* K12 MG1655 (accession no. U00096); P.A., *Pseudomonas aeruginosa* PA01 (accession no. AE004091); F.N., *Fusobacterium nucleatum* ATCC 25586 (accession no. AE009951). These bacteria can be divided into three respiratory patterns: Facultative anaerobes, A.P., P.M., M.S., E.C.; Aerobe, P.A.; Anaerobe, F.N.
