# Supplementary material for: Genome Biology of Actinobacillus pleuropneumoniae JL03, an Isolate of Serotype 3 Prevalent in China
Source: PLoS One. 2008 Jan 16;3(1):e1450. doi: 10.1371/journal.pone.0001450 (PMC2175527; doi:10.1371/journal.pone.0001450)
Supplement: Table S2 — Crucial enzymes and functional proteins involved in metabolic pathways of A. pleuropneumoniae strain JL03 (0.13 MB DOC) [file pone.0001450.s002.doc]

**Table S2**. Crucial enzymes and functional proteins involved in metabolic pathways of *A. pleuropneumoniae* strain JL03

| Name | CDS no. | Putative function |
| --- | --- | --- |
| *pssA* | APJL0044 | CDP-diacylglycerol—serine O-phosphatidyltransferase |
| *dAK1* | APJL0082 | dihydroxyacetone kinase |
| *dhaK* | APJL0083 | dihydroxyacetone kinase |
| *nrfA* | APJL0100 | nitrate reductase, cytochrome c552 |
| *nrfB* | APJL0101 | nitrate reductase, cytochrome-C type protein |
| *nrfC* | APJL0102 | nitrate reductase, Fe-S protein |
| *nrfD* | APJL0103 | nitrate reductase, transmembrane protein |
| *grk* | APJL0143 | glycerate kinase |
| *nqrA* | APJL0151 | NADH-ubiquinone oxidoreductase subunit A |
| *nqrB* | APJL0152 | NADH-ubiquinone oxidoreductase subunit B |
| *nqrC* | APJL0153 | NADH-ubiquinone oxidoreductase subunit C |
| *nqrD* | APJL0154 | NADH-ubiquinone oxidoreductase subunit D |
| *nqrE* | APJL0155 | NADH-ubiquinone oxidoreductase subunit E |
| *nqrF* | APJL0156 | NADH-ubiquinone oxidoreductase subunit F |
| *rnfA* | APJL0166 | Na+-translocating NADH-quinone oxidoreductase |
| *rnfB* | APJL0167 | ferredoxin II, iron sulfur protein |
| *rnfC* | APJL0168 | iron-sulfur binding NADH dehydrogenase |
| *rnfD* | APJL0170 | Na-translocating NADH-quinone reductase |
| *rnfG* | APJL0171 | electron transport complex protein |
| *rnfE* | APJL0172 | Na-translocating NADH-quinone reductase |
| *pykA* | APJL0188 | pyruvate kinase II |
| *pgpA* | APJL0204 | phosphatidylglycerophosphatase A |
| *dxs* | APJL0208 | 1-deoxyxylulose-5-phosphate synthase |
| *gpmB* | APJL0235 | phosphoglycerate mutase |
| *pgsA* | APJL0285 | CDP-diacylglycerol--glycerol-3-phosphate 3-phosphatidyltransferase |
| *cydA* | APJL0308 | cytochrome D ubiquinol oxidase, subunit I |
| *cydB* | APJL0309 | cytochrome D ubiquinol oxidase, subunit II |
| *ppc* | APJL0355 | phosphoenolpyruvate carboxylase |
| *glpF* | APJL0392 | glycerol uptake facilitator protein |
| *glpA* | APJL0398 | anaerobic glycerol-3-phosphate dehydrogenase, subunit A |
| *glpB* | APJL0399 | anaerobic glycerol-3-phosphate dehydrogenase, subunit B |
| *glpC* | APJL0400 | anaerobic glycerol-3-phosphate dehydrogenase, subunit C |
| *dxr* | APJL0428 | 1-deoxy-D-xylulose 5-phosphate reductoisomerase |
| *lpxD* | APJL0433 | UDP-3-O-(3-hydroxymyristoyl) glucosamine N-acyltransferase |
| *cdsA* | APJL0437 | phosphatidate cytidylyltransferase |
| *pgpB* | APJL0441 | phosphatidylglycerophosphatase B |
| *gapA* | APJL0460 | glyceraldehyde 3-phosphate dehydrogenase |
| *sucD* | APJL0478 | succinyl-CoA synthetase alpha chain |
| *sucC* | APJL0479 | succinyl-CoA synthetase beta chain |
| *sucB* | APJL0481 | dihydrolipoamide succinyltransferase |
| *sucA* | APJL0482 | 2-oxoglutarate dehydrogenase E1 component |
| *manB* | APJL0642 | phosphomannomutase |
| *dld* | APJL0685 | D-lactate dehydrogenase |
| *lpdA* | APJL0774 | dihydrolipoamide dehydrogenase |
| *aceF* | APJL0775 | dihydrolipoamide s-acetyltransferase |
| *aceE* | APJL0776 | pyruvate dehydrogenase E1 component |
| *prsA* | APJL0778 | ribose-phosphate pyrophosphokinase |
| *ispE* | APJL0779 | 4-diphosphocytidyl-2-C-methyl-D-erythritolkinase |
| *pckA* | APJL0805 | phosphoenolpyruvate carboxykinase |
| *ispD* | APJL0807 | 2C-methyl-D-erythritol4-phosphatecytidylyltransferase |
| *ispF* | APJL0808 | 2C-methyl-D-erythritol 2,4-cyclodiphosphate synthase |
| *gpmA* | APJL0840 | 2,3-bisphosphoglycerate-dependent phosphoglycerate mutase |
| *lpxL* | APJL0912 | lipid A acyltransferase |
| *wbbJ* | APJL0918 | acetyltransferase |
| *tktA* | APJL1003 | transketolase |
| *adh2* | APJL1029 | alcohol dehydrogenase/acetaldehyde dehydrogenase |
| *arsC* | APJL1105 | arsenate reductase |
| *plsB* | APJL1126 | glycerol-3-phosphate acyltransferase |
| *eno* | APJL1132 | enolase |
| *pfkA* | APJL1143 | phosphofructokinase |
| *cytB562* | APJL1148 | soluble cytochrome b562 |
| *pji* | APJL1156 | glucose-6-phosphate isomerase |
| *adh1* | APJL1221 | alcohol dehydrogenase class III |
| *ispG* | APJL1198 | 1-hydroxy-2-methyl-2(E)-butenyl 4-diphosphatesynthase |
| *fbaA* | APJL1261 | fructose-bisphosphate aldolase |
| *pgk* | APJL1262 | phosphoglycerate kinase |
| *ndh* | APJL1282 | NADH dehydrogenase |
| *mdh* | APJL1309 | malate dehydrogenase |
| *gnd* | APJL1319 | 6-phosphogluconate dehydrogenase |
| *nagB1* | APJL1323 | glucosamine-6-phosphateisomerase |
| *zwf* | APJL1324 | glucose-6-phosphate 1-dehydrogenase |
| *cytB* | APJL1351 | Ni/Fe-hydrogenase 2 b-type cytochrome subunit |
| *fbp* | APJL1420 | fructose-1,6-bisphosphatase |
| *psd* | APJL1432 | phosphatidylserine decarboxylase |
| *napC* | APJL1457 | nitrate/TMAO reductase, membrane-bound tetraheme cytochrome c subunit |
| *napB* | APJL1458 | nitrate reductase cytochrome c-type subunit |
| *napH* | APJL1459 | polyferredoxin |
| *napG* | APJL1460 | ferredoxin 2 |
| *napA* | APJL1461 | anaerobic dehydrogenase |
| *napD* | APJL1462 | NapD protein |
| *napF* | APJL1463 | ferredoxin-type protein |
| *rpiA* | APJL1475 | ribose 5-phosphate isomerase A |
| *plsC* | APJL1513 | 1-acyl-sn-glycerol-3-phosphate acyltransferase |
| *elaA* | APJL1530 | acetyltransferase |
| *gpsA* | APJL1536 | glycerol-3-phosphate dehydrogenase |
| *ispH* | APJL1546 | hydroxymethyl butenyl pyrophosphate reductase |
| *frdD* | APJL1553 | fumarate reductase |
| *frdC* | APJL1554 | fumarate reductase |
| *frdB* | APJL1555 | fumarate reductase iron-sulfur protein |
| *frdA* | APJL1556 | fumarate reductase flavoprotein subunit |
| *rbsK2* | APJL1704 | sugar kinase |
| *fumC* | APJL1792 | fumarate hydratase |
| *rpe* | APJL1856 | D-ribulose-phosphate-3 epimerase |
| *cysI* | APJL1881 | sulphite reductase beta subunit |
| *cysJ* | APJL1882 | sulfite reductase flavoprotein subunit |
| *cysN* | APJL1883 | sulfate adenylate transferase subunit 1 |
| *cysD* | APJL1884 | sulfate adenylate transferase subunit 2 |
| *cysH* | APJL1885 | 3'-phosphoadenosine 5'-phosphosulfate sulfotransferase |
| *lldD* | APJL1891 | L-lactate dehydrogenase |
| *tpiA* | APJL1972 | triosephosphate isomerase |
| *adh3* | APJL2006 | alcohol dehydrogenase |
| *aldA* | APJL2061 | aldehyde dehydrogenase |
