# Supplementary material for: Genome Biology of Actinobacillus pleuropneumoniae JL03, an Isolate of Serotype 3 Prevalent in China
Source: PLoS One. 2008 Jan 16;3(1):e1450. doi: 10.1371/journal.pone.0001450 (PMC2175527; doi:10.1371/journal.pone.0001450)
Supplement: Table S3 — Genes encoding proteins involved in iron metabolism of A. pleuropneumoniae JL03 compared with the homologous proteins from three representative genomes within Pasteurellaceae (0.19 MB DOC) [file pone.0001450.s003.doc]

**Table S3**. Genes encoding proteins involved in iron metabolism of *A. pleuropneumoniae* JL03 compared with the homologous proteins from three representative genomes within *Pasteurellaceae*.

| *A. pleuropneumoniae* JL03 | | | *A. pleuropneumoniae* L20 | | | *H. ducreyi* 35000HP | | | *P. multocida* Pm70 | | |
| --- | --- | --- | --- | --- | --- | --- | --- | --- | --- | --- | --- |
| CDS no. | Name | Function | CDS no. | Name | Identity | CDS no. | Name | Identity | CDS no. | Name | Identity |
| APJL0076 | *tonB2* | TonB energy transducing protein | APL0076 | *tonB2* | 100 | HD0327 | *tonB* | 70.97 | Pm1188 | *tonB* | 47.95 |
| APJL0077 | *exbD2* | biopolymer transport protein | APL0077 | *exbD2* | 100 | HD0328 | *exbD* | 91.47 | Pm1187 | *exbD* | 69.6 |
| APJL0078 | *exbB2* | biopolymer transport protein | APL0078 | *exbB2* | 100 | HD0329 | *exbB* | 87.07 | Pm1186 | *exbB* | 63.95 |
| APJL0113 | *hemE* | uroporphyrinogen-III decarboxylase | APL0112 | *hemE* | 98.87 |  |  | - | Pm1734 | *uroD* | 85.59 |
| APJL0128 | *yfeD* | iron (chelated) transport system membrane protein | APL0127 | *yfeD* | 100 | HD1024 | *yfeD* | 81.16 | Pm0397 | *yfeD* | 67.15 |
| APJL0129 | *yfeC* | iron (chelated) transport system membrane protein | APL0128 | *yfeC* | 100 | HD1025 | *yfeC* | 87.1 | Pm0398 | *yfeC* | 75.64 |
| APJL0250 | *tbpB2* | transferrin-binding protein 2 | APL0245 | | 99.82 |  |  | - |  |  |  |
| APJL0251^a^ | *tbpA2’* | transferrin-binding protein 1 | APL0246 | | - |  |  |  |  |  |  |
| APJL0280 | *yfeB* | iron (chelated) transporter, ATP-binding protein | APL0271 | | 100 | HD1817 | *yfeB* | 87.92 | Pm0399 | *yfeB* | 76.92 |
| APJL0282^a^ | *yfeA’* | iron ABC transporter, periplasmic-binding protein | APL0272 | *yfeA* | - | HD1816 | *yfeA* |  | Pm0400 | *yfeA* |  |
| APJL0286 | *frpB* | iron-regulated outer membrane protein | APL0276 | *frpB* | 100 |  |  |  |  |  |  |
| APJL0552 | *afuA2* | iron(III) transport system substrate-binding protein | APL0563 | *afuA_2* | 97.96 |  |  | - | Pm0203 |  | 63.37 |
| APJL0553 | *afuB2* | iron(III) transport system permease protein | APL0564 | *afuB_2* | 96.25 |  |  |  | Pm0956 | *afuB_1* | 63.62 |
| APJL0554^a^ | *cirA’* | outer membrane receptor proteins, mostly Fe transport | APL0565 | *cirA* | - |  |  | - |  |  |  |
| APJL0665 | - | high-affinity Fe^2+^/Pb^2+^ permease | APL0670 | | 98.54 |  |  | - |  |  |  |
| APJL0714 | - | ABC-type enterochelin transport system, periplasmic component | APL0714 | | 100 |  |  | - |  |  |  |
| APJL0715 | - | iron(III) transport system permease protein | APL0715 | | 99.66 |  |  | - |  |  |  |
| APJL0716 | - | iron(III) transport system permease protein | APL0716 | | 98.97 |  |  | - |  |  |  |
| APJL0717 | - | iron(III) transport system ATP-binding protein | APL0717 | | 99.6 | HD0572 | *potA* | 32.91 | Pm0128 | *fecE* | 39.21 |
| APJL0856 | *bcp* | bacterioferritin comigratory protein | APL0846 | *bcp* | 96.15 | HD1667 | *bcp* | 85.71 | Pm1052 | *bcp* | 73.03 |
| APJL0866 | *hbpA1* | heme-binding protein A | APL0855 | *hbpA* | 99.44 | HD0215 | *hbpA* | 58.89 | Pm0592 | *hbpA* | 60.35 |
| APJL0930 | *irp* | iron-regulated outer membrane protein | APL0919 | *irp* | 99.87 |  |  | - | Pm1428 |  | 47.16 |
| APJL0939 | *iscA* | iron-binding protein | APL0929 | *iscA* | 100 | HD1084 |  | - | Pm0320 |  | 85.05 |
| APJL0977^a^ | - | large exoprotein involved in heme utilization adhesion |  |  | - |  |  | - |  |  |  |
| APJL0979 | - | large exoprotein involved in heme utilization adhesion | APL0959 | | 97.96 |  |  |  |  |  |  |
| APJL0980 | - | hemolysin activation/secretion protein |  |  | - |  |  |  |  |  |  |
| APJL1065 | *hgbA* | hemoglobin and hemoglobin haptoglobin-binding protein 4 | APL1047 | *hgbA* | 99.89 | HD2025 | *hgbA* | 51.17 | Pm0300 |  | 58.14 |
| APJL1066 | *hugZ* | heme iron utilization protein | APL1048 | *hugZ* | 99.42 | HD0620 | *hugZ* | 87.28 | Pm0299 |  | 77.84 |
| APJL1085 | *ftnA* | ferritin-like protein 1 | APL1069 | *ftnA* | 98.81 | HD1754 | *ftnA* | 84.52 | Pm0666 | *rsgA_1* | 76.4 |
| APJL1086 | *ftnB* | ferritin-like protein 2 | APL1070 | *ftnB* | 100 | HD1755 | *ftnB* | 79.11 | Pm0667 | *rsgA_2* | 67.68 |
| APJL1182 | - | predicted iron-dependent peroxidase | APL1162 | | 96.64 |  |  | - | Pm0455 |  | 58.16 |
| APJL1231 | *fur* | ferric uptake regulation protein | APL1218 | *fur* | 99.33 | HD0367 | *fur* | 93.96 | Pm0352 | *fur* | 70.27 |
| APJL1312 | - | iron-regulated outer membrane protein | APL1299 | | 99.43 |  |  |  |  |  |  |
| APJL1387 | *ccmD* | heme exporter protein D | APL1369 | *ccmD* | 98.08 |  |  | - | Pm0008 | *ccmD* | 53.06 |
| APJL1388 | *ccmC* | heme exporter membrane protein | APL1370 | *ccmC* | 99.59 | HD0789 | *ccmC* | 87.55 | Pm0007 | *ccmC* | 71.07 |
| APJL1389 | *ccmB* | heme exporter membrane protein | APL1371 | *ccmB* | 100 | HD0787 | *ccmB* | 92.73 | Pm0006 | *ccmB* | 78.54 |
| APJL1390 | *ccmA2* | cytochrome c-type biogenesis ATP-binding protein | APL1372 | *ccmA* | 100 | HD0786 | *ccmA* | 78.95 | Pm0005 | *ccmA* | 57.14 |
| APJL1416 | *afuA1* | AfuA protein | APL1446 | *afuA* | 99.13 |  |  | - | Pm0953 | *afuA_1* | 81.21 |
| APJL1417 | *afuB1* | ABC-type thiamine transport system, permease components | APL1447 | *afuB* | 98.69 |  |  |  | Pm0956 | *afuB_1* | 75.63 |
| APJL1418 | *afuC* | ferric transport protein | APL1448 | *afuC* | 99.43 | HD0572 | *potA* | 53.14 | Pm0957 | *afuC* | 83.57 |
| APJL1583 | *hemL* | glutamate-1-semialdehyde aminotransferase | APL1555 | *hemL* | 99.77 |  |  |  | Pm0462 | *hemL* | 80.52 |
| APJL1597 | *tbpA1* | transferrin-binding protein 1 | APL1567 | *tbpA* | 98.15 |  |  |  |  |  |  |
| APJL1598 | *tbpB1* | transferrin-binding protein 2 | APL1568 | *tbpB* | 59.88 |  |  | - |  |  |  |
| APJL1599 | *exbD* | biopolymer transport protein | APL1569 | *exbD* | 100 |  |  |  | Pm0969 | *tolR* | 34.06 |
| APJL1600 | *exbB* | biopolymer transport protein | APL1570 | *exbB* | 100 | HD1777 | *tolQ* | 34.67 | Pm0970 | *tolQ* | 33.04 |
| APJL1601 | *tonB1* | periplasmic protein | APL1571 | *tonB1* | 99.59 |  |  |  |  |  |  |
| APJL1776 | - | Fe^2+^-trafficking protein | APL1740 | | 100 | HD2003 |  | 86.52 | Pm1320 |  | 89.66 |
| APJL1827 | - | Fe^3+^-hydroxamate transport system, periplasmic component | APL1791 | | 99.73 |  |  |  |  |  |  |
| APJL1828 | - | Fe(III) dicitrate ABC transporter, permease | APL1792 | | 84.06 |  |  |  | Pm1308 | *hemU* | 36.39 |
| APJL1829 | - | hemin import ATP-binding protein | APL1793 | | 98.82 | HD1826 | *modC* | 32.02 |  |  |  |
| APJL1831 | - | Fe^3+^-hydroxamate transport system, periplasmic component | APL1795 | | 98.37 |  |  |  |  |  |  |
| APJL1922 | - | outer membrane receptor proteins, mostly Fe transport |  |  | - | HD0646 |  | 66.19 |  |  |  |
| APJL1983 | *hemH* | protoheme ferro-lyase | APL1937 | *hemH* | 97.81 |  |  |  | Pm0789 | *hemH* | 60.75 |
| APJL2000^a^ | - | hemoglobin receptor precursor | APL1047 | *hgbA* | |  |  |  | Pm0741 |  | 58.92 |
| APJL2036 | *hemB* | delta-aminolevulinic acid dehydratase | APL1988 | *hemB* | 100 |  |  |  | Pm1692 | *hemB* | 78.21 |
| APJL2060 | *hbpA2* | heme-binding protein A | APL2010 | *hbpA* | 98.35 | HD0215 | *hbpA* | 82.94 | Pm0592 | *hbpA* | 75.69 |
| APJL2063 | *fhuC* | Fe^3+^-siderophores transport systems, ATPase components | APL2013 | *fhuC* | 99.56 |  |  |  | Pm0128 | *fecE* | 38.8 |
| APJL2064 | *fhuD* | ferrichrome-binding periplasmic protein | APL2014 | | 97.85 |  |  |  |  |  |  |
| APJL2065 | *fhuB* | ferrichrome uptake protein | APL2015 | *fhuB* | 96.15 |  |  |  |  |  |  |
| APJL2066 | *fhuA* | outer membrane ferric hydroxamate receptor | APL2016 | | 38.44 |  |  |  |  |  |  |

^a^ These CDS nos. stand for pseudogenes impaired by mutation in *A. pleuropneumoniae* JL03. The homologous genes with >30% identity were selected out.
