# Supplementary material for: Genome Biology of Actinobacillus pleuropneumoniae JL03, an Isolate of Serotype 3 Prevalent in China
Source: PLoS One. 2008 Jan 16;3(1):e1450. doi: 10.1371/journal.pone.0001450 (PMC2175527; doi:10.1371/journal.pone.0001450)
Supplement: Table S4 — Genes encoding proteins associated with virulence factors (apx toxins, proteases, and urease) in A. pleuropneumoniae JL03 (0.06 MB DOC) [file pone.0001450.s004.doc]

**Table S4**. Genes encoding proteins associated with virulence factors (apx toxins, proteases, urease) in *A. pleuropneumoniae* JL03

| CDS no. | Name | Function |
| --- | --- | --- |
| APJL0009 | *sohB* | serine protease |
| APJL0121 | *prc* | tail-specific protease |
| APJL0247 | *argC* | N-acetyl-gamma-glutamyl-phosphate reductase |
| APJL0248 | *argB* | acetylglutamate kinase |
| APJL0394 | *lon* | ATP-dependent protease LA |
| APJL0696 | *argA* | amino-acid acetyltransferase |
| APJL0744 | *degS* | protease DegS |
| APJL0896 | *proB* | glutamate 5-kinase |
| APJL0931 | *tonB3* | periplasmic protein |
| APJL0967 | *apxIIA* | hemolysin A |
| APJL0968 | *apxIIC* | hemolysin-activating lysine-acyltransferase |
| APJL1057 | *htpX* | putative protease |
| APJL1101 | *argE* | acetylornithine deacetylase |
| APJL1103 | *clpB* | ATP-dependant Clp protease chain B |
| APJL1139 | *gcp* | putative sialylglycoprotease |
| APJL1279 | *sppA* | protease IV, signal peptide peptidase |
| APJL1291 | *clpX* | ATP-dependent Clp protease ATP-binding subunit |
| APJL1292 | *clpP* | ATP-dependent Clp protease proteolytic subunit |
| APJL1344 | *apxIIID* | RTX-III toxin determinant D |
| APJL1345 | *apxIIIB* | RTX-III translocation ATP-binding protein |
| APJL1346 | *apxIIIA* | RTX-III toxin determinant A |
| APJL1347 | *apxIIIC* | hemolysin-activating lysine-acyltransferase |
| APJL1644 | *ureH* | urease accessory protein UreH |
| APJL1645 | *ureG* | urease accessory protein UreG |
| APJL1646 | *ureF* | urease accessory protein UreF |
| APJL1647 | *ureE* | urease accessory protein UreE |
| APJL1648 | *ureX* | UreX protein |
| APJL1649 | *ureC* | urease alpha subunit |
| APJL1650 | *ureB* | urease beta subunit |
| APJL1651 | *ureA* | urease gamma subunit |
| APJL1652 | *utp* | urea transport protein |
| APJL1771 | *hslU* | ATP-dependent protease, ATPase subunit |
| APJL1772 | *hslV* | ATP-dependent protease |
| APJL1926 | *ptrA* | protease III |
| APJL1933 | *lonH* | lon protease |
| APJL1960 | *pepO* | metallopeptidase |
| APJL1998 | *proA* | gamma-glutamyl phosphate reductase |
