# Supplementary material for: Genome Biology of Actinobacillus pleuropneumoniae JL03, an Isolate of Serotype 3 Prevalent in China
Source: PLoS One. 2008 Jan 16;3(1):e1450. doi: 10.1371/journal.pone.0001450 (PMC2175527; doi:10.1371/journal.pone.0001450)
Supplement: Text S1 — Supplementary Materials and Methods (0.04 MB DOC) [file pone.0001450.s005.doc]

**Supporting Text**

**Materials and Methods**

**Genome annotation and analysis**. Glimmer, RBSFinder [1], and FgenesB (http://softberry.com) were used to identify putative protein coding sequences (CDSs), and the final results were verified by ARTEMIS [2]. BLASTP [3] was used for automated annotation based on sequence similarity in conjunction with searching the definitive CDSs set against Kyoto Encyclopedia of Genes and Genomes (KEGG) database, SwissProt database and NCBI Non-redundant protein database. The cluster of orthologous groups of proteins (COGs) database was applied to identify family classification of predicted proteins [4]. Manual revision was carried out for clarity and for satisfying annotations to the current literatures.

The (5S, 16S and 23S) ribosomal RNA (rRNA) genes were predicted by RNAmmer 1.1a server [5]. The transfer RNA (tRNA) genes were identified by tRNAscan-SE v1.23 [6]. Transfer-messenger RNA genes (tmRNAs) were predicted by using BRUCE [7]. Transmembrane helices (TMHs) and signal peptides of CDSs were found by the packages TMHMM 2.0 and SignalP 3.0 respectively [8, 9]. The program SIGI-HMM was used to predict genomic islands and the putative donor of each individual alien gene [10]. The software package GenomeViz [11] was used to visualize the genome organization trait and specific genetic characteristics of JL03.

Ortholog proteins for two genome’s CDSs were defined if protein identity was above 60%, alignment coverage above 80% and E value below 1e-50. For the JL03 genome, repetitive proteins were determined when protein identity was above 95% and E value was below 1e-100. Repetitive DNA elements were defined as those that had at least 300 bp with cutoff value of 1e-10.

Whole genome sequences of *A. pleuropneumoniae* L20 of serotype 5b, *P. multocida* Pm70, *H. ducreyi* 35000HP, *H. somnus* 129PT and Escherichia coli K12 MG1655 were retrieved from GenBank database. Contigs of unfinished genomes of *A. pleuropneumoniae* 4074 of serotype 1 (accession no. AACK00000000) and *M. haemolytica* A1 (AASA00000000) were used as reference in genomic comparison. The genomic sequence comparison was carried out by the program GenomeComp [12].

**Nucleotide sequence accession number**. The genome sequence of JL03 is available from GenBank with the assigned accession number CP000687.

1. Delcher A, Harmon D, Kasif S, White O, Salzberg S (1999) Improved microbial gene identification with GLIMMER. Nucleic Acids Res 27: 4636-4641.
2. Rutherford K, Parkhill J, Crook J, Horsnell T, Rice P, et al. (2000) Artemis: Sequence visualization and annotation. Bioinformatics 16: 944-945.
3. Altschul SF, Gish W, Miller W, Myers EW, Lipman DJ (1990) Basic local alignment search tool. J Mol Biol 215: 403-410.
4. Tatusov RL, Galperin MY, Natale DA, Koonin EV (2000) The COG database: a tool for genome-scale analysis of protein functions and evolution. Nucleic Acids Res 28: 33-36.
5. Lagesen K, Hallin P, Rodland EA, Staerfeldt HH, Rognes T, et al. (2007) RNAmmer: consistent and rapid annotation of ribosomal RNA genes. Nucleic Acids Res 35: 3100-3108.
6. Lowe TM, Eddy SR (1997) tRNAscan-SE: a program for improved detection of transfer RNA genes in genomic sequence. Nucleic Acids Res 25: 955-964.
7. Laslett D, Canback B, Andersson S (2002) BRUCE: a program for the detection of transfer-messenger RNA genes in nucleotide sequences. Nucleic Acids Res 30: 3449-3453.
8. Krogh A, Larsson B, von Heijine G, Sonnhammer EL (2001) Predicting transmembrane protein topology with a hidden Markov model: application to complete genomes. J Mol Biol 305: 567-580.
9. Bendtsen JD, Nielsen H, von Heijne G, Brunak S (2004) Improved prediction of signal peptides: SignalP 3.0. J Mol Biol 340: 783-795.
10. Waack S, Keller O, Asper R, Brodaq T, Damm C, et al. Score-based prediction of genomic islands in prokaryotic genomes using hidden Markov models. (2006) BMC Bioinformatics 7: 142.
11. Ghai R, Hain T, Chakraborty T (2004) GenomeViz: visualizing microbial genomes. BMC Bioinformatics 5: 198.
12. Yang J, Wang JH, Yao ZJ, Jin Q, Shen Y, et al. (2003) GenomeComp: a visualization tool for microbial genome comparison. J Microbiol Meth 54: 423-426.
